# Utilization of Machine Learning for the Differentiation of Positional NPS Isomers with Direct Analysis in Real Time Mass Spectrometry

**DOI:** 10.1021/acs.analchem.1c04985

**Published:** 2022-03-17

**Authors:** Jennifer L. Bonetti, Saer Samanipour, Arian C. van Asten

**Affiliations:** † Van’t Hoff Institute for Molecular Sciences, University of Amsterdam, P.O. Box 94157, Amsterdam 1090 GD, The Netherlands; ‡ Virginia Department of Forensic Science, Norfolk, Virginia 23606, United States; § Co van Ledden Hulsebosch Center (CLHC), Amsterdam Center for Forensic Science and Medicine, 1098 XH Amsterdam, The Netherlands

## Abstract

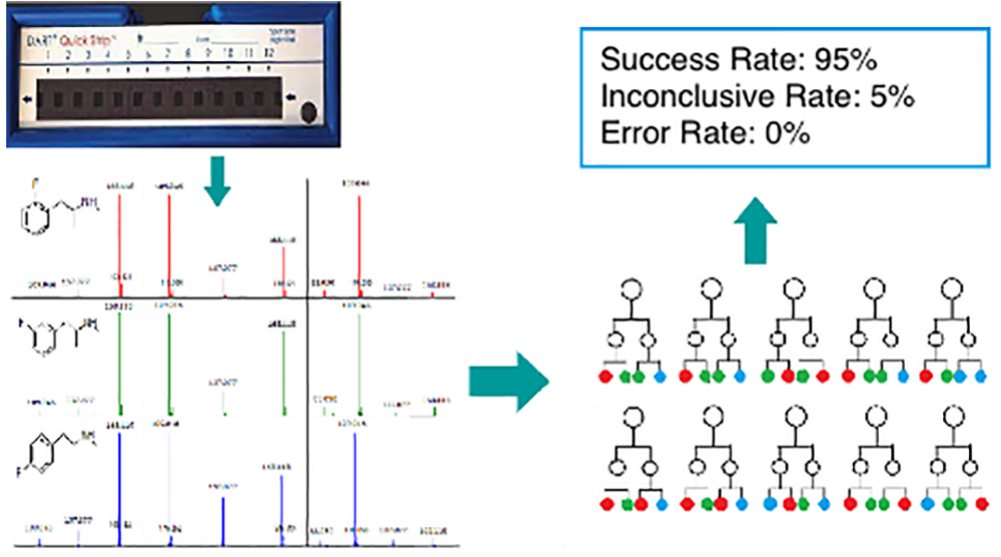

The differentiation of positional isomers is a well established analytical challenge for forensic laboratories. As more novel psychoactive substances (NPSs) are introduced to the illicit drug market, robust yet efficient methods of isomer identification are needed. Although current literature suggests that Direct Analysis in Real Time–Time-of-Flight mass spectrometry (DART-ToF) with in-source collision induced dissociation (is-CID) can be used to differentiate positional isomers, it is currently unclear whether this capability extends to positional isomers whose only structural difference is the precise location of a single substitution on an aromatic ring. The aim of this work was to determine whether chemometric analysis of DART-ToF data could offer forensic laboratories an alternative rapid and robust method of differentiating NPS positional ring isomers. To test the feasibility of this technique, three positional isomer sets (fluoroamphetamine, fluoromethamphetamine, and methylmethcathinone) were analyzed. Using a linear rail for consistent sample introduction, the three isomers of each type were analyzed 96 times over an eight-week timespan. The classification methods investigated included a univariate approach, the Welch *t* test at each included ion; a multivariate approach, linear discriminant analysis; and a machine learning approach, the Random Forest classifier. For each method, multiple validation techniques were used including restricting the classifier to data that was only generated on one day. Of these classification methods, the Random Forest algorithm was ultimately the most accurate and robust, consistently achieving out-of-bag error rates below 5%. At an inconclusive rate of approximately 5%, a success rate of 100% was obtained for isomer identification when applied to a randomly selected test set. The model was further tested with data acquired as a part of a different batch. The highest classification success rate was 93.9%, and error rates under 5% were consistently achieved.

## Introduction

The previous decade has been marked by a considerable increase in the complexity and diversity of novel psychoactive substances (NPSs) arriving on the illicit drug market. As of December 2020, over 1000 new NPSs have been reported, nearly double the number reported by the end of 2019 and nearly eight times as many as in 2009.^1^ This onset of chemical complexity in casework has presented a substantial challenge to forensic chemists as techniques that have been the gold standard for decades (gas chromatography coupled with mass spectrometry (GC-MS) combined with nonspecific presumptive testing methods) are typically incapable of differentiating highly similar NPS structures. A common struggle is the conclusive identification of positional ring isomers, compounds whose structures only differ in the precise location of a substituent on an aromatic ring. This challenge is of particular interest because positional NPS isomers are not always controlled at the same level. In The Netherlands, 4-methylmethcathinone (4-MMC) is a List I compound and thus controlled as a “hard drug”, whereas its positional ring isomer 3-MMC was only recently classified as a List II chemical (“soft drug”)^2^ and 2-MMC is currently not regulated at all.

Many different approaches have been investigated for improving the ease with which forensic experts can confidently differentiate positional ring isomers. Some recent successful approaches include GC-IRD,^3^ GC-VUV,^4−6^ UPLC,^7^ product ion spectrometry,^8^ and infrared ion spectroscopy.^9^ Since GC-MS is so commonly utilized in the testing of novel psychoactive substances in forensic laboratories, the analysis of these data has been improved using chemometric analysis as a means of enhancing the technique’s isomeric differentiation capabilities.^5,10−19^ Some of these studies used a univariate approach where each *m*/*z* fragment is analyzed separately with an unequal variance *t* test.^10−12^ Other studies have seen success with a multivariate approach such as principal component analysis (PCA) followed by linear discriminant analysis (LDA),^5,13^ LDA with different variable selection techniques,^14^ hierarchal clustering analysis,^15^ and canonical discriminant analysis.^16,17^ Another study used PCA solely as a means to highlight which *m*/*z* ions to further investigate for differentiation.^18^ Despite the success of these currently available techniques, many require significant analysis time, either through additional sample preparation such as derivatization^19^ or simply the more substantial time required for a typical GC analysis, particularly since a blank, sample, and standard would each require a separate injection. In contrast, the minimal sample preparation required for Direct Analysis in Real Time–Time-of-Flight mass spectrometry (DART-ToF) in combination with its rapid analysis time make it an underutilized resource for tackling the NPS challenge.

Given its high throughput and intrinsic selectivity, DART-ToF has been successfully introduced into forensic laboratories.^20−22^ The DART source was introduced in 2004,^23^ and its many forensic applications have been extensively reviewed.^24^ Several publications note the instrument’s ability to differentiate positional drug isomers, particularly when combined with in-source collision induced dissociation (is-CID), typically introduced through function switching of the Orifice 1 voltage.^25,26^ However, these publications do not touch on aromatic ring isomers in particular, focusing instead on positional isomers that involve more substantial structural changes such as hydrocodone vs codeine^25^ and cathinones with changes to the carbon backbone.^26^ These structural changes result in clear visual differences in the DART-ToF spectra, allowing the forensic expert to confidently assign the correct structure. These notable differences are absent from the DART spectra of aromatic ring isomers, making differentiation via visual examination challenging, if not impossible. Further complicating the issue is the presence of ambient ionization which, while largely responsible for many of the strengths of the instrument, results in a considerable amount of variation from one analysis to the next. Therefore, if NPS ring isomer differentiation is to be attempted based on DART-ToF data, more advanced data analysis techniques must be investigated.

The results of this study showcase the improved ability of the Random Forest classifier over other more common chemometric methods to highlight small but reproducible differences in DART-ToF spectra for the purpose of NPS isomer differentiation. Three sets of positional ring isomers were studied, including fluoroamphetamine (FA) and fluoromethamphetamine (FMA), both of which have *para*-isomers that have recently gained popularity in The Netherlands,^27,28^ and the methylmethcathinones (MMCs). The structures are provided in Figure S1 of the Supporting Information. To our knowledge, this work is the first reported attempt to differentiate ring isomers of psychoactive substances with DART-ToF.

## Experimental Section

### Reagents and Materials

The nine isomers were purchased as primary standards in the form of hydrochloride salts from Cayman Chemical (Ann Arbor, MI) with the exception of 4-MMC, which was purchased from Sigma-Aldrich (St. Louis, MO). The isomer identity of each standard was confirmed using Fourier-transform infrared spectroscopy (FTIR). The solvents used were GC grade methanol and multipurpose use chloroform from Honeywell B&J Brand (Muskegon, MI). Solutions were prepared as the hydrochloride salt at a base concentration of 0.1 mg/mL. The phenethylamines were prepared in chloroform, while the cathinones were prepared in methanol per recommendations from Ciallella et al.^29^ All solutions were stored at −20 °C when not in use.

The polyethylene glycol (PEG) 600 (Tokyo Chemical Industry, Co. Portland, OR) calibrator solution was prepared at a concentration of 0.5 mg/mL in methanol. As described in the seminal DART-ToF publication, this solution results in a spectrum with fragments of known accurate mass across the *m*/*z* range and can therefore be used to calibrate the mass axis of the instrument.^23^ A drug mix consisting of approximately 161:8:32 μg/mL of a cocaine base (Sigma-Aldrich), methamphetamine HCl (Sigma-Aldrich), and nefazadone (Alfa Aesar, Haverhill, MA) in methanol was utilized for quality assurance (QA) to ensure successful calibration for all analytical runs, which is consistent with current casework procedures.^21^

### Data Acquisition

Data were acquired using a Direct Analysis in Real Time (Model DART SVP, IonSense, Saugus MA) AccuToF (Model JMS-T100LP, JEOL USA, Peabody MA) system equipped with a linear rail according to the analytical parameters listed in Table S1. The linear rail was utilized with QuickStrip cards from IonSense to minimize spectral variation caused by sample introduction.

Over a period of 8 weeks in early 2021, four sample QuickStrip cards were analyzed per isomer type on a weekly basis. Each QuickStrip card had 12 separated mesh locations for sample deposition. The QuickStrip card setup is shown in Table S2. For the calibrator, drug mix, and solvent blank, 3 μL spots were deposited onto the appropriate mesh locations using a Gilson (Middleton, WI) M25 pipette. Each drug compound was run in triplicate on each QuickStrip card by placing 3 μL of the 0.1 mg/mL drug solutions in each sample location for two of the four QuickStrip cards and 6 μL per sample location for the other two QuickStrip cards. In total, this resulted in 96 analyses per compound, or 288 analyses per isomer type. Each QuickStrip card, containing nine separate drug analyses, had an elapsed run time of between 6 and 6.5 min. Therefore, the entire data set for each isomer type was generated within 3.5 h of instrumental run time over the analysis period.

Each Orifice 1 voltage utilized via the function switching setting produced a “total ion chronogram” (response vs time). All spectra were collected using the MassCenter software (Jeol USA, Peabody, MA) by averaging the instrument response across the width of the appropriate peak. In the case of the drug samples, the spectra were background subtracted by subtracting the average response over the solvent blank region of the chronogram. All spectra were centroided with an abundance threshold of 120. The 30 V spectrum of the PEG calibrator solution for each QuickStrip card was used to calibrate the mass axis for the data file, and the 30 V QA drug mix spectrum was used to confirm successful calibration with an acceptance criteria of ±5 mDa for each test compound.^21^ Sample spectra were collected from the 30, 60, and 90 V total ion chronograms and saved as .jsp files. A Perl script was used to convert all of the drug spectra for each isomer type into a single .csv file containing spectra for all three Orifice 1 voltages in terms of raw abundance in *m*/*z* bins of 0.025 Da width. The bins were named for the upper limit. For example, the 109.050 bin contained abundance values for *m*/*z* 109.025–109.050 Da.

While the primary data for this study were acquired in early 2021, an external validation set of FMA data was generated, originally for exploratory purposes, using 1.0 mg/mL solutions in the latter half of 2020. This data set included 11 QuickStrip cards of data, set up in the same manner as Table S2 and using the same instrument parameters as seen in Table S1. The first three QuickStrip cards used 10 μL depositions, and the latter eight used 3 μL depositions. It was eventually determined that this concentration was too high with a potential risk of carryover between the samples, which is why this data set was not included in the main study. However, due to these differences from the main data set, both in terms of time between analyses and concentrations used, this data set was ultimately used to further test the robustness of the Random Forest classifier.

### Data Analysis

#### Calculations

All variable selection, normalization, and subsequent data analysis was performed using R (R version 4.0.0, Rstudio Version 1.2.5042, Boston, MA). The packages utilized were dplyr, stringr, xlsx, ggplot2, ggpubr, randomForest, ggrepel, grid, gridExtra, gtable, MASS, reshape2, and matrixStats. The github repository containing all code is available online.^30^

#### Variable Selection and Normalization

To limit the data set to the *m*/*z* bins of interest, several steps were taken. To start, *m*/*z* bins were not specific to a given voltage, meaning that a single sample had three separate spectra of data, one for each voltage. Each *m*/*z* bin was investigated to determine if it met the “percent abundance threshold“ set for the data set. To do this, each spectrum was temporarily normalized to a base peak value of 100%. Any *m/z* bin for which at least 50 spectra did not meet the set percent abundance threshold was discarded. For the MMC isomers, the percent abundance thresholds were set at 1% and 10%. For the FA and FMA isomers, the 1% threshold resulted in minimal to no additional bins when compared to the 10% threshold. Therefore, the percent abundance thresholds were set at 0.3% and 10% for these two isomer types. This base peak normalization was only performed to identify *m/z* bins above the percent abundance threshold. As alternative normalization techniques were explored, the original raw abundance values were utilized in the remaining data analysis steps.

The reduced data set was then separated by voltage for further variable reduction. It was common for some *m*/*z* bins to reach the overall percent abundance threshold but not substantially contribute to all voltages. Each voltage was examined to identify the most relevant *m*/*z* bins. A step-by-step illustration of this process can be found in Figure S3. The final resulting *m*/*z* bins for each voltage for each isomer type are shown in Table S3.

Two types of normalization were investigated. In both cases, spectra were normalized per voltage. The first type was referred to as “ion current” in which the abundance for each *m/z* bin was normalized by the sum of the abundance values for all retained *m/z* bins in the same voltage. In other words, each abundance was normalized by the total ion current generated only by the selected *m/z* bins. The second normalization was referred to as “vector length”. In this case, the abundance values for a given voltage were considered as a vector, and this vector was normalized to a unit vector by dividing by its length. Therefore, the abundance for each *m*/*z* bin was normalized by the square root of the sum of the abundance values of all *m*/*z* bins at the same voltage. For both normalization procedures, only contributions from the final selected *m*/*z* bins were considered. An example of each normalization calculation procedure is shown in Table S4. After normalization, the *m*/*z* bins from all three voltages were combined into one row of data per sample, with one normalized abundance value per *m*/*z* bin and voltage combination. In this manner, each *m*/*z* bin/voltage combination was one separate variable.

To preprocess the external validation data set, the same *m*/*z* bins that were identified for the main FMA data set were retained. Again, both vector length and ion current normalization methods were studied.

#### Multiway Analysis of Variance

A multiway Analysis of Variance (ANOVA) was performed on each of the final normalized *m*/*z* bins based on isomer identity, the week the analysis was performed, and the volume used for sample deposition. For the three isomer types, this was performed separately for both normalization types as well as for both percent abundance thresholds studied.

#### Univariate Analysis

The first method of analysis performed was based on the statistical comparison of electron impact (EI) mass spectral data as introduced by Bodnar Willard et al.^31^ and applied in numerous studies^10−12^ in which the unequal variance *t* test (Welch test) is applied at each *m*/*z* fragment. In this manner, it is determined whether the mean abundance at any given *m*/*z* fragment is distinguishable between an unknown sample and a known isomer. The comparison of an unknown to its correct isomer identity should result in a higher number of indistinguishable *m*/*z* fragments than the comparison of the unknown sample to an incorrect isomer identity. To apply this method, a leave-one-sample-out cross-validation approach was employed where “one” refers to each QuickStrip card’s worth of data. For each triplicate of data from the excluded QuickStrip card, the Welch test was performed at each *m*/*z* bin to compare the test triplicate to the remaining data for each known isomer separately. The number of indistinguishable *m*/*z* bins was counted for each comparison. This entire analysis was conducted 20 times, with a randomly selected confidence level between 90 and 99.999% and a randomly selected normalization technique.

To test the accuracy of using this analysis as a classification method, it was investigated whether there exists a threshold of indistinguishable *m*/*z* bins that would only be reached by a comparison of a test sample with its correct isomer identity. Receiver Operator Characteristic (ROC) curves were generated by plotting the false positive rate (FPR) vs the true positive rate (TPR) based on an increasing number of indistinguishable *m*/*z* bins for each replicate analysis. In addition, ROC curves were also prepared using the average FPR and TPR of the 20 replicates in order to assess the general robustness and accuracy of this type of classification.

An alternate method was attempted in which the results (number of indistinguishable *m*/*z* bins) for the comparisons of the test triplicate to each of the three isomers were compared to one another. In this case, rather than a set number of indistinguishable *m*/*z* bins needing to be achieved, the test set was classified as belonging to whichever isomer had the highest number of indistinguishable *m*/*z* bins provided the minimum difference when compared to the other two isomers exceeded a selected threshold. If this threshold difference was not met, the result was considered inconclusive.

For both methods, a second analysis was performed wherein the training data set was only made up of the QuickStrip cards that were analyzed on the same day as the excluded test QuickStrip card. The accuracy of each *m*/*z* bin was assessed by dividing the sum of the true positives and true negatives by the total number of comparisons.

#### Multivariate Classification

The multivariate method applied was Linear Discriminant Analysis. As discussed above, multivariate methods including LDA have been used successfully for the differentiation of EI/MS data. In addition, Easter and Steiner used both PCA and LDA when validating the isomer differentiation potential of DART-ToF for pharmaceutical confirmation.^25^ LDA was performed using the normalized abundance values for all included *m*/*z* bins with no additional variable reduction step. LDA assumes a multivariate normal distribution and equivalent variance for all classes to calculate the posterior probability that an unknown sample belongs to each class. The prior probability that an unknown sample could belong to a particular class given no experimental data was set to be equal for all isomer identities. The accuracy of this supervised classification method was assessed using leave-one-sample-out cross-validation as well as randomly separating the data set into 80% training and 20% test sets. For both validation methods, two types of conclusions were tested. First, the removed sample(s) were classified by simply assigning the sample(s) to the isomer with the highest calculated posterior probability. Subsequently, the posterior probabilities were compared to an acceptance criteria of sequentially increasing thresholds (0.50–0.95, stepwise by 0.05). If the highest calculated posterior probability for the classification of an excluded sample to an isomer was above the threshold, then this assignment was compared to the actual isomer identity to determine if the classification was correct or erroneous. If the posterior probability was below the threshold, the result was considered inconclusive.

The validation procedure described above was also applied to the data collected on a single day. As with the full data set analysis, conclusions were based on both the isomer resulting in the highest posterior probability as well as by incrementally increasing thresholds in comparison to the posterior probabilities of the excluded sample(s).

The influence of each *m*/*z* bin on both linear discriminant functions was determined by the scaling coefficient for each variable. A high absolute coefficient value indicated that the given *m*/*z* bin had a high impact on the transformation onto that particular axis.

#### Machine Learning Algorithm

Finally, a machine learning technique was utilized. The Random Forest classifier, introduced by Breiman in 2001, utilizes a large number of uncorrelated decision trees acting as an ensemble to result in a more robust classifier than any single decision tree by itself.^32^ The lack of correlation is partly ensured through bootstrapping, wherein the number of samples in the data set for a given decision tree in the random forest is equal to the number of samples in the original data set. However, due to random sampling-with-replacement, the exact makeup of the data set will differ between trees. In addition, for any given node of a decision tree, the variables available for selection are selected from a limited random subset of the original variables. Once the “forest” of decision trees is generated, unknown samples are classified based on which class is the assigned result for the highest proportion of decision trees. For this study, default parameters in the random Forest package in R were utilized in order to explore the feasibility of applying this technique for the purpose of isomer classification. Therefore, the number of decision trees used was 500, and the number of variables in the subsets at each node was equal to the square root of the total number of variables.

To assess the robustness of the classification, “out-of-bag” samples were used in which each sample was analyzed using the decision trees which were unaffected by this specific sample. Each of these trees gave a classification result, and the proportion of trees assigning the sample to each isomer was determined. Typically, the samples would be classified as whichever isomer was assigned the highest proportion of decision trees. To avoid making conclusions in cases where there was not a clear distinction between the isomers, the highest resulting proportion of trees was instead evaluated against an acceptance criterion of incrementally increasing thresholds (0.50–0.95, stepwise by 0.05) before reaching a conclusion. If the threshold was met, the classification was compared to the known isomer identity to determine if the classification was either correct or flawed. If the highest proportion of trees was below the threshold, the result was considered inconclusive.

For both LDA and the Random Forest classifier, thresholds were introduced after the models were generated and thus did not change the models themselves or the output of the classifiers. They only served to determine whether a classification met an acceptance criterion in order to make a conclusion. Thus, while increasing thresholds could reduce the type I (false positive) error rate (and/or true positive ”success” rate) of a series of analyses, they could not serve to change the resulting classification of a sample from one isomer to another, only from a potentially erroneous conclusion to an inconclusive result.

In addition to out-of-bag samples, the data set was also randomly separated into 80% training and 20% test sets. The test set samples were then analyzed by all 500 decision trees, and the same threshold comparison was performed.

As with the univariate and multivariate classifiers, the analysis was also performed using only samples analyzed on the same day. Out-of-bag samples were again utilized as well as selecting each QuickStrip card as a test set and generating the forest using the remaining QuickStrip cards for a specific day.

Permutation of the variables was applied as a sensitivity analysis in order to assess the impact of each *m*/*z* bin.^32^ After determining the out-of-bag accuracy rate for the full data set (classifications based solely on the isomer assignment with the highest proportion of trees), the abundance values in each *m*/*z* bin were randomly shuffled among samples. After each permutation, the out-of-bag samples were classified again, and the accuracy of the classifier was re-established. The importance of each variable was determined by how much its permutation affected the accuracy and is reported as the Mean Decrease Accuracy (MDA).

To assess the robustness of the classifier to the removal of low impact variables, a series of 12-fold cross validations were performed with removal of the least important variables. Half of the variables were removed sequentially until a single variable remained.

## Results and Discussion

### ANOVA, Distribution and Stability of Isomer Abundance

**Figure 1:**
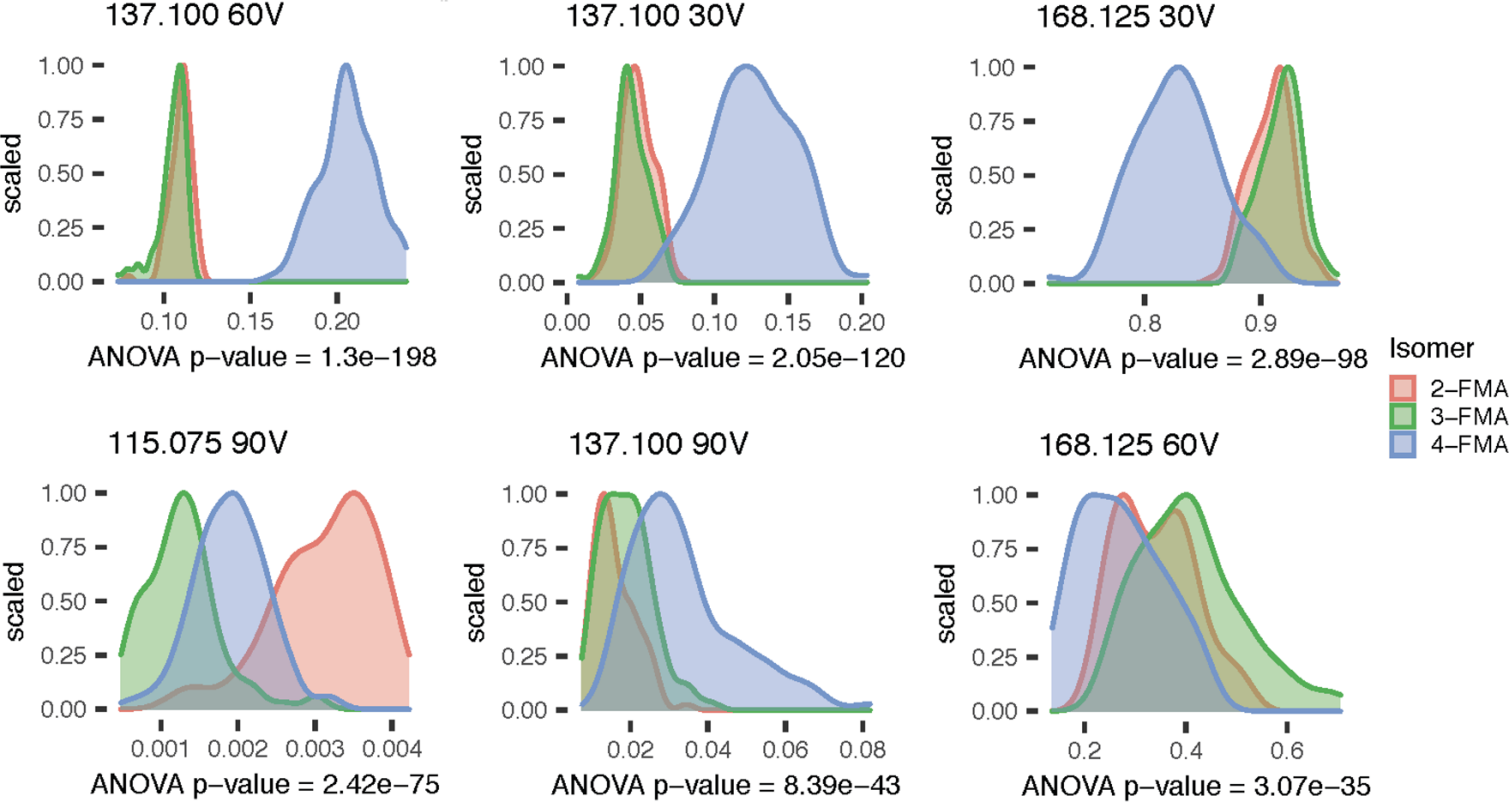
Density distribution of the top six most discriminating *m*/*z* bins for FMA isomers using 0.3% abunndance threshold and ion current normalization. Top six determined by increasing ANOVA *p* value based on isomer identity.

To illustrate the challenge in differentiating the DART-ToF spectra of these isomers, an example is shown in Figure S2, which was generated using the function switching technique and showing the data at three different voltages, the two highest of which (60 and 90 V) produce fragmentation patterns due to is-CID. The isomer spectra are visually indistinguishable, which is why three chemometric techniques were applied in an effort to statistically determine whether any latent differences exist that can be used for isomer classification. An example of the density distribution for six *m*/*z* bins of the FMA data set can be found in Figure 1. Mean abundance values with associated standard deviation for the lowest percent abundance threshold data sets with ion current normalization are shown in Tables S6–S8.

In order to assess whether statistical differences existed in the data set, a multiway ANOVA was performed on each *m*/*z* bin based on isomer identity, analysis week, deposition volume, and the interactions between those three factors. The results of the multiway ANOVA showed that for nearly all *m*/*z* bins, the isomer identity had a significant effect on the mean abundance (*p* < 0.05). For example, for the FA data set with a 0.3% abundance threshold, the average *p* value was 3.63 × 10^–2^ with a standard deviation of 1.78 × 10^–1^ when ion current normalization was applied. Only one *m*/*z* bin (109.050 30 V) showed a nonsignificant difference based on isomer identity (*p* = 8.71 × 10^–1^). If removed, the average *p* value for this data set was 1.57 × 10^–5^ with a standard deviation of 5.42 × 10^–5^. Additional ANOVA results based on isomer identity, week, and volume are shown in Table S5.

The sample deposition volume had a significant effect on the mean abundance for approximately half of the *m*/*z* bins for all three isomer types. This proportion of *m*/*z* bins was seen regardless of the percent abundance threshold applied, suggesting that it was not only the low abundance *m*/*z* bins that showed significant differences based on deposition volume.

The analysis week had a significant impact on the mean abundance for most of the bins for all isomers, showing similar *p* value ranges and number of significant *m*/*z* bins to the isomer identity analysis. In addition, the interaction between isomer identity and the week of analysis also frequently had a significant effect on the mean abundance. To investigate this further, plots were generated for each bin showing the change in mean abundance per isomer over time with error bars showing the standard deviation. Although there were some changes week-to-week, when these plots were produced as a function of an accumulating data set (week 1; weeks 1 and 2; weeks 1, 2, and 3; etc.) the changes seemed to stabilize for most bins and most isomers after 4–5 weeks. As the sampling area of the instrument is open to the atmosphere, small changes in relative ion abundance are expected when performing analyses on different days due to small changes in the immediate environment. The collection of data over multiple days seems to ensure this variability is normalized, which will enhance the changes in abundance values due to isomer identity. An example of a week-to-week plot and its corresponding accumulating data set plot is shown in Figure S4 and Figure S5, respectively.

### Univariate Classification

**Figure 2:**
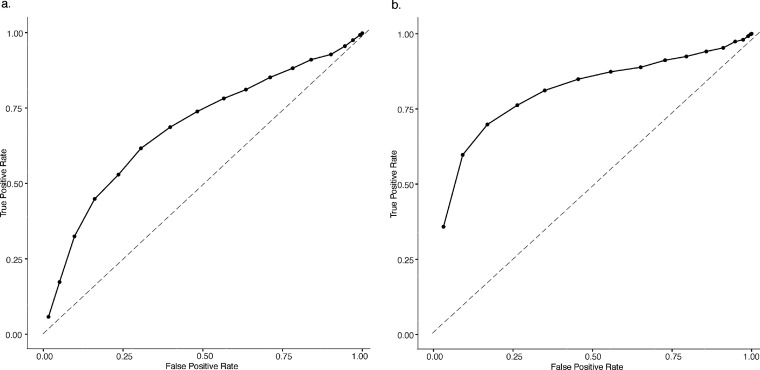
Receiver operator characteristic curves for an average of 20 replicates of Welch test analysis of FMA isomers, using a 0.3% abundance threshold for *m*/*z* bin selection using (a) full data set (AUC = 0.7131) and (b) same day restricted data set (AUC = 0.8346). Dotted line approximating a random classifier of AUC = 0.5 is shown.

As seen in an example in Figure 2 as well as in Figures S6 and S7, the Welch test method performed better than a random classifier but was ultimately not robust or accurate enough for use in a forensic laboratory. For each type of isomer, applying the lower percent abundance threshold consistently resulted in the best classification results as this produced a higher number of variables (*m*/*z* bins) for the comparison. The procedure of comparing the test QuickStrip card to a data set containing only other QuickStrip cards analyzed on the same day did perform marginally better than using the full data set, but overall the selectivity was still insufficient for casework. The results for the individual replicates of the Welch test analysis did not vary substantially from one another.

### Multivariate Classification

**Figure 3:**
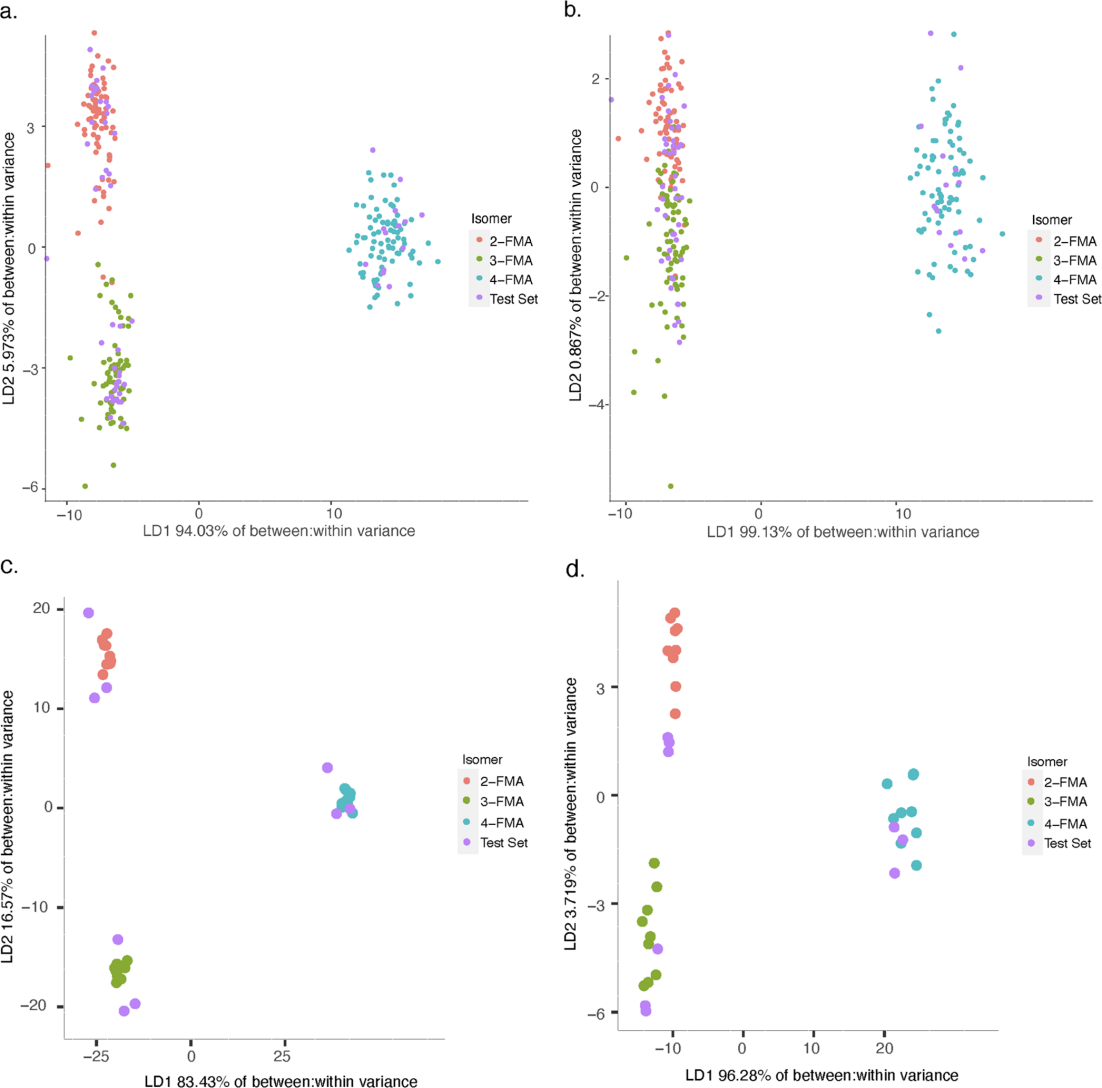
Linear discriminant analysis scores plots for training vs test sets of FMA data set using ion current normalization for (a) full data set 0.3% threshold, (b) full data set 10% threshold, (c) same day analysis (first week, first QuickStrip card as test set) 0.3% threshold, (d) same day analysis (first week, first QuickStrip card as test set) 10% threshold.

Linear Discriminant Analysis was more successful than the Welch test at discriminating the isomers, albeit with some limitations. As seen in Figure 3, as well as in Figures S8 and S9, the data sets with the lower percent abundance threshold and therefore more *m*/*z* bins to use for LDA showed more separation between the most similar isomers. For the FA and FMA isomers, the most challenging isomer pair was the 2- and 3-isomers (*ortho* and *meta*), whereas for the MMC isomers, the 3- and 4- (*meta* and *para*) isomers were most difficult to differentiate. For the MMC isomers with the 1% abundance threshold, the same-day analysis resulted in an error due to the bin containing *m*/*z* 129.075 90 V data producing a within-class variance below the default allowed tolerance (1.0 × 10^–8^). This bin was therefore removed for this analysis.

Following the example set by Kranenburg et al., the likelihood ratios (LR) for the conclusions were calculated by dividing the highest posterior probability by the second highest posterior probability.^5^ Calibration of LR values has been the focus of many recent forensic studies.^33−37^ However, for this study, no thorough validation of the likelihood ratios was conducted due to the suspected overfitting of the LDA model. Thus, the following LR results should be used for indicative purposes only. For the example shown in Figure 3, the LR values ranged from 60 to 1.72 × 10^120^, with the LR of 60 arising from the only sample that was misclassified. However, when the percent abundance threshold was raised to 10%, there was less differentiation between the FMA isomers, particularly 2-FMA and 3-FMA. Using the same normalization, there were now nine misclassifications (LR range from 1.1 to 86) with considerable LR overlap with the successful classifications (1.2–9.5 × 10^113^). Twenty-eight of the correct classifications (approximately 48% of the total conclusions) resulted in LR values that overlapped with those resulting from erroneous conclusions.

In some instances, same-day analysis showed excellent separation and 100% successful classification of the test set samples without the use of thresholds to rule out inconclusive samples, even with the higher percent abundance threshold data sets. However, this success was not consistent among all QuickStrip cards and all weeks, with several test sets giving error rates as high as 44.44% (four of nine samples on the test set QuickStrip card were misclassified). The misclassifications appeared to be due to a combination of overfitting of the LDA model and insufficient separation of especially similar isomers. In addition, some of the misclassified samples produced very high likelihood ratios. For example, within the FMA data set using the 0.3% abundance threshold and ion current normalization, a 3-FMA sample from the third QuickStrip card analyzed on week five was misclassified as 2-FMA with a likelihood ratio of 7.2 × 10^36^. These high error rates were only partially reduced with the use of thresholds, as discussed later. In all cases, any separation between the most similar isomers was only seen along the second linear discriminant function axis, which frequently explained a limited fraction of the between-to-within class variance present in the data set. This suggests a high degree of correlation between the variables, which was indeed found to exist as illustrated through the heat maps in Figures S10–S12.

### Machine Learning and Classification of External Validation Data

**Figure 4:**
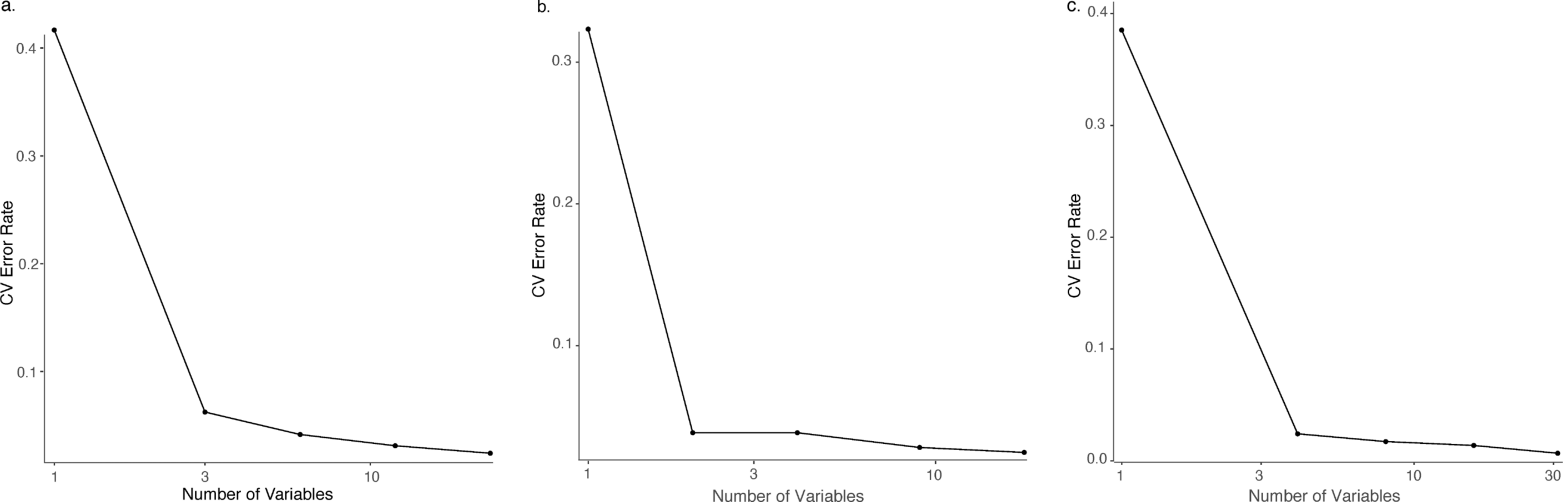
Random Forest 12-fold cross-validation error rates observed after removing the least important variables sequentially. The *x* axis is shown on a logarthmic scale. All examples are from data sets with the lowest percent abundance threshold applied and ion current normalization. (a) FA, (b) FMA, (c) MMC.

As seen in Figure 4, even without the use of thresholds to reduce error rates, the Random Forest classifier already performed quite well and maintained a low error rate for all three isomer types even as the number of variables was decreased. For the FA and FMA data sets with the higher percent abundance threshold, these error rates were higher and more affected by the variable reduction. This suggests that there is important discriminating information present in the lower abundance *m*/*z* bins.

When thresholds were incorporated, and the data set was split into 80% training and 20% test sets, the Random Forest classifier frequently achieved a 0% error rate associated with inconclusive rates of approximately 5% or lower, as seen in Table 1, particularly for the lower percent abundance threshold data sets. The phenethylamines in particular were more affected by the higher percent abundance thresholds, with the FMA 10% abundance threshold data set showing a test set error rate of 14% at a proportion of decision trees threshold of 50% when using an ion current normalization. However, that error rate was reduced to 5.2% at a decision tree threshold of 60%, increasing the inconclusive rate to 19%. In contrast, the highest error rate achieved with the MMC test set using the higher percent abundance threshold was only 5.2%, and the same data set achieved a 0% error rate at a proportion of decision trees threshold of 65% with an inconclusive rate of 14%. For all isomers, this suggests that low abundance ions have an impact on the ability of the method to differentiate the isomers.

**Table 1:** Classification Results for Random Forest 20% Test Sets Using Ion Current Normalization and Lowest Percent Abundance Threshold

		threshold									
		0.50	0.55	0.60	0.65	0.70	0.75	0.80	0.85	0.90	0.95
FMA	success	0.983	0.966	0.948	0.948	0.914	0.896	0.862	0.758	0.603	0.362
	inconclusive	0	0.017	0.052	0.052	0.086	0.104	0.138	0.242	0.397	0.638
	error	0.017	0.017	0	0	0	0	0	0	0	0
FA	success	0.983	0.983	0.948	0.948	0.914	0.896	0.810	0.810	0.620	0.310
	inconclusive	0	0	0.052	0.052	0.086	0.104	0.190	0.190	0.380	0.690
	error	0.017	0.017	0	0	0	0	0	0	0	0
MMC	success	1	0.983	0.983	0.983	0.983	0.948	0.914	0.775	0.620	0.431
	inconclusive	0	0.017	0.017	0.017	0.017	0.052	0.086	0.225	0.380	0.0.569
	error	0	0	0	0	0	0	0	0	0	0

Since the Random Forest classifier gave the lowest consistent error rates for the main isomer data sets, the external validation data set was used to challenge the model. In addition to utilizing the thresholds of proportions of decision trees to determine whether a conclusion was made for each sample, it was also investigated whether making only one conclusion per triplicate rather than one per sample would further improve the accuracy of this method. This is possibly more in line with how forensic laboratories would integrate this method into their current workflow.

The first method tested was that all three samples in the triplicate had to result in the same conclusion. If they did not, the result was inconclusive. If they did, the average proportion of decision trees among all three samples was compared to the thresholds. The second method allowed for only two of the three samples in the triplicate to reach the same conclusion (majority voting). If at least two samples gave the same isomer, the proportions of trees for the identifying samples were averaged and compared to the thresholds. Finally, the third method of reaching a conclusion was to only record a result for the sample in the triplicate which amassed the highest proportion of trees. This sample’s proportion of trees was then compared to the thresholds and used to reach a conclusion.

Despite the differences between the main data set and the external validation data set, the Random Forest classifier built with the main data set was still successful in most instances at classifying the external validation data set. This success was typically further improved when using the triplicate data of each sample to arrive at a final conclusion as shown in Table 2. Interestingly, for most proportions of trees thresholds, the data set with the higher abundance threshold actually showed somewhat reduced error rates, although this frequently could be attributed to increased inconclusive rates. It is also important to note here that the external validation data set was only made up of 99 samples, or 33 triplicates. So, a 3% error rate when only one conclusion is made per triplicate is equivalent to only one misclassified triplicate.

**Table 2:** Random Forest Classification Results for External Validation FMA Data Set −0.3% Ion Current (Bottom Three Sections Show One Conclusion Made Per Triplicate Analysis)

		threshold									
		0.50	0.55	0.60	0.65	0.70	0.75	0.80	0.85	0.90	0.95
all samples	success	0.828	0.657	0.515	0.444	0.384	0.313	0.040	0	0	0
	inconclusive	0.081	0.283	0.465	0.535	0.606	0.677	0.960	1	1	1
	error	0.091	0.061	0.020	0.020	0.010	0.010	0	0	0	0
all 3 match	success	0.727	0.576	0.485	0.394	0.364	0.333	0.030	0	0	0
	inconclusive	0.242	0.394	0.485	0.606	0.636	0.667	0.970	1	1	1
	error	0.030	0.030	0.030	0	0	0	0	0	0	0
2 or more	success	0.939	0.758	0.606	0.394	0.364	0.333	0.030	0	0	0
	inconclusive	0	0.182	0.364	0.606	0.636	0.667	0.970	1	1	1
	error	0.061	0.061	0.030	0	0	0	0	0	0	0
highest	success	0.879	0.788	0.667	0.576	0.424	0.394	0.091	0	0	0
	inconclusive	0	0.091	0.242	0.333	0.545	0.576	0.909	1	1	1
	error	0.121	0.121	0.091	0.030	0.030	0	0	0	0	0

### Comparison of the Three Classification Methods

For all three chemometric methods, the introduction of thresholds served to reduce the error rates by labeling the less convincing classifications as inconclusive. However, while in some cases the LDA error rate decreased, in many instances changing the thresholds did not affect the results to the same extent as observed for the Welch test and the Random Forest classifier. This is likely due to some degree of overfitting, resulting in extremely high posterior probabilities even for incorrect classifications. Although LDA often resulted in low error rates, those rates could not be reduced to zero through threshold adjustment as was often feasible for the Random Forest classifier.

The Welch *t* test typically gave the highest error rates of the three classifiers, and while this could be decreased to 0% with increasing thresholds, it always came at the cost of an extremely high inconclusive rate (>80%). For casework, this would mean that in a vast majority of the cases, follow-up analyses using other techniques would be required, effectively nullifying the benefits of using DART-ToF. Due to the speed of the instrumental technique and the ease with which multiple samples can be analyzed at one time, the inconclusive rates achieved by the Random Forest classifier, even when the higher percent abundance threshold data sets were used, would be manageable in a forensic casework laboratory. Plots showing a visual representation of these comparisons for the full data sets are shown in Figures S13–S15. The same day (excluded QuickStrip card treated as test set) analysis comparisons can be found in Figures S16–S18.

### Variable Importance and Chemical Reasoning

Although each method naturally lends itself to a different means of assessing the contribution of *m*/*z* bin to the classification, the importance of each variable can still be investigated across all three methods. Plots showing variable importance per analysis method for the three isomers are depicted in Figures S19–S21.

For the FA and FMA isomers, the bins at *m*/*z* 137.100 were consistently important variables, in all voltages, for the Random Forest classifier. This fragment, likely C_9_H_10_F, occurs through the removal of the amine from the protonated molecule, either through rearrangement and subsequent loss of NH_3_ (FA) or through the bond dissociation between the nitrogen and its α carbon (FMA).^38^ For both phenethylamines, the 4-isomer (*para*) was easiest to distinguish from the remaining isomers, and this loss is one likely reason. The mean abundance of the bin at *m*/*z* 137.100 for all voltages was considerably higher for the *para* isomer for both FA and FMA and for both normalization methods. The presence of the fluorine as an electron-withdrawing group in the *para* position allows for stabilizing conjugation of this protonated fragment which makes this loss more favorable for this isomer.^39^ The difference between the mean abundance of the isomers in this bin is less prominent when the Orifice 1 voltage is increased to 90 V as this induces collisions of sufficient energy for subsequent fragmentation. Fittingly, for the FMA isomers, this fragment was influential for the first linear discriminant function which differentiated the *para*-isomer from the others, but not the second function (which was most effective at distinguishing the more similar isomers).

The 137.100 fragment was not as influential for either linear discriminant function for the FA isomers. Instead, another variable that was influential for all three classification methods was the *m*/*z* 135.075 (C_9_H_8_F) at both 60 and 90 V. This fragment likely is formed by the removal of H_2_ from the 137.100 fragment. The normalized mean abundances of all three isomers for this fragment at 90 V were separated from one another by more than one standard deviation with the highest abundance seen in the *para* isomer and the lowest in the *ortho* isomer. Interestingly, the [M + H – HF]^+^ fragment that is highly abundant in the chemical ionization mass spectra of fluoroamphetamines^8^ was not present in high enough abundance to exceed even the lowest percent abundance threshold.

For both the Random Forest classifier and the second linear discriminant function, the most important variable for the FMA isomers was the bin containing *m*/*z* 115.075 90 V data. This fragment was also particularly influential in the second linear discriminant function for the FA isomers and somewhat influential in the Random Forest classifier as well as the first linear discriminant function. This fragment’s accurate mass indicates a C_9_H_7_ ion, possibly a phenyl cyclopropyl ion, formed during the is-CID occurring at the highest Orifice 1 voltage. The ortho effect likely plays a role here as the mean abundance of this *m*/*z* bin was higher for the *ortho* isomer than the mean abundance of the other two isomers by more than one standard deviation for both normalization methods and both the FA and FMA isomers.

For the MMC isomers, the bins at *m*/*z* 129.075 (C_10_H_9_) and *m*/*z* 128.075 (C_10_H_8_) provided a large influence on all three classification methods. In addition, the *m*/*z* 130.075 (C_10_H_10_) for both 60 and 90 V heavily influenced the second linear discriminant function. These fragments all likely formed from the loss of a methylamine from the protonated molecule (with varying amounts of unsaturation) and a water molecule. For both normalization methods, the *ortho*-isomer had a higher mean abundance for these three fragments compared with the mean abundances of the same fragments for the other two isomers, frequently differing by more than one standard deviation. This suggests the presence of an ortho effect favoring these fragmentation pathways through the formation of a six-member ring as a transition state.^40,41^ This ortho effect seems particularly prevalent for the 129.075 fragment, which may be why it drives the first linear discriminant function, which easily differentiates the *ortho* isomer from the other two.

Interestingly, one of the most influential *m*/*z* bins for the Random Forest classifier of the MMC isomers was the 160.125 (C_11_H_14_N) fragment in the 60 V Orifice 1 voltage. This peak likely originated due to the loss of a water molecule as a result of a rearrangement reaction along with the formation of an azirine-containing ion as proposed by Franski et al.^42^ The normalized mean abundance of this variable did differ between the three isomers, but frequently not by more than one standard deviation. Although the aromatic substitution is not involved in this rearrangement, one might expect this fragment to have the highest mean abundance for the *ortho* isomer due to the ortho effect, but actually the *para* isomer showed the highest mean abundance, followed by *ortho* and then the *meta* isomer. This might be due to the fragment forming more readily for the *ortho* isomer (as it is more abundant in the 30 V data) and subsequently dissociating into the 128.075, 129.075, and 130.075 fragments.

For all three isomer types, when the lower percent abundance threshold was utilized, the low abundance variables typically provided a high amount of influence on the classification models. This demonstrates the important contributions of the low abundance *m*/*z* bins in the correct identification of the NPS ring isomer. This suggests that while variable reduction is important to reducing the effects of mixture interference, it is beneficial to retain more *m*/*z* bins than just those that may be visually identified as prominent peaks.

## Conclusions and Future Work

While the benefits and strengths of the DART-ToF instrument are plentiful, it is well understood that ambient sample ionization makes use of this data for positional ring isomer differentiation challenging due to the high variation between analyses and the absence of chromatographic separation prior to MS analysis. This study has shown that while all three types of chemometric classification methods show some success in tackling this challenge, a machine learning technique, such as the Random Forest classifier, appears to be most well-suited to handle this analytical problem by effectively utilizing minor differences in the mass spectra to assign the correct isomeric form. The two normalization techniques showed similar performance, although the ion current method seemed to slightly outperform the vector length procedure, particularly in terms of the inconclusive rate at which a 0% error rate was achieved. However, this improvement in performance was minimal, and both methods of normalization are worth considering in future studies.

There are some limitations when it comes to incorporating this rapid and novel classification method into the analytical scheme for a forensic casework laboratory. The most difficult challenge to overcome is interference from possible mixture constituents. Typically, these classes of NPS are not found in mixtures with the same frequency as other common drugs of abuse, but it is still worth investigating. It is the hope of the authors that by restricting the analysis and normalization to only *m*/*z* fragments that are relevant to the isomer, as determined from the analysis of primary standards, extraneous fragments due to diluents or cutting agents may be ignored. In addition, a key benefit to the Random Forest classifier is that each tree is unique and does not include all variables. Both of these features may allow this model to still successfully differentiate NPS ring isomers in case mixtures. However, future studies must be conducted including casework samples to determine if the presence of common cutting agents would impact the fragmentation of the isomer itself. This type of work has been conducted on fentanyl and some fentanyl analogues, and minimal competitive ionization effects were seen, though it is important to note that this study focused on ionization for detection, not necessarily the reproducibility of fragmentation patterns in the presence of mixtures.^43^

Since mixtures are a well-known challenge for DART-ToF in many instances; there has been some work performed in sample cleanup techniques such as solid-phase microextraction^44,45^ and other reproducible sample introduction techniques such as thermal desorption.^43,46^ It is possible that in the presence of challenging mixtures, the combination of appropriate sample clean up steps and data analysis tools such as those presented here could still result in successful NPS isomer classification. However, it is unlikely that this method could be used if a sample contains a mixture of two or more different isomeric forms (for example 2-FA and 4-FA). Although this is rare in casework, it is possible, and it needs to be investigated whether this method would be capable of determining that such a mixture is present or whether an erroneous conclusion would be reached.

Other challenges in moving this work from proof-of-concept into use in forensic casework involve possible sample-to-sample variation. In this study, only one primary standard for each isomer was utilized, so while highly pure it is unknown if there may be impurities from the synthesis that could affect the analysis when compared to street samples. Similarly, differences in concentration could affect the results. However, this is why two different deposition volumes were included in the data set. Sample concentration much higher than used here would likely result in sample carryover, while sample concentrations lower would show a low TIC signal. Both instances would cause an experienced analyst to adjust the concentration to a more suitable level.

An additional limitation that needs to be investigated is whether this model could be utilized to identify isomers that were analyzed on a different DART-ToF instrument than the one used to create the classifier model. For this study, all samples were analyzed on the same instrument, but it is the hope that eventually other laboratories could compare their data to one central, robust data set to identify their unknown samples without the need to create their own data sets. In practice, this would involve the laboratories analyzing their own standards to compare against the model data set to ensure correct classification is achieved, and subsequently an unknown sample could be confidently analyzed. Future work will involve the creation of a simple user interface to accommodate the use of a centralized model. In order for this to be feasible, another important challenge to investigate is model stability over time for the various DART-ToF setups. As the instruments are used and serviced, does excessive variation build up over time that would impede the success of the model?

While these challenges have yet to be addressed, the success seen here with data taken all on one day suggests that if such a robust, centralized data set is not feasible, a quick, smaller data set could be easily generated in the course of the analysis and still produce rapid, successful ring isomer differentiation. Although the instrumental time required to generate the entire data set for each isomer was much smaller than for a comparably sized GC-MS data set, the time required for spectral acquisition after analysis is a potential limitation. Currently the software that is typically used with this instrument does not have the functionality to automatically extract multiple spectra from the total ion chronogram generated for each Orifice 1 voltage. There may however be commercially available software packages that can be explored to expedite this process. Once the model is constructed (either a large centralized model or a smaller lab-specific model), the authors anticipate that such a model can be used directly and for a prolonged period of time. This means that model creation is a one-time investment. The combination and preprocessing of the data is conducted through a computer script^30^ and is performed nearly instantaneously immediately before comparison to the classification models. It is the hope that, with the proposed future step of a centralized model and user interface, this process would be made even simpler. In that case, isomer identification with DART-MS could be established in a similar time frame as a typical library search and in a very high throughput compared to GC-MS.

## Supporting Information Available

DART method parameters, isomer structures, Quickstrip card setup, example FMA DART 30/60/90 V spectra, variable selection walkthrough, final retained *m*/*z* bins, normalization example, ANOVA results, FMA plots showing week-to-week changes, plots showing accumulating FMA data set changes over time, mean abundances for low percent abundance threshold data sets, ROC curves for FA and MMC, LDA scores plots for FA and MMC, correlation heat maps, comparison plots for three methods, variable importance comparison per method (PDF)

## Notes

The data underlying this study are openly available in Figshare at https://doi.org/10.21942/uva.19346771.
