# Supplementary material for: Utilization of Machine Learning for the Differentiation of Positional NPS Isomers with Direct Analysis in Real Time Mass Spectrometry
Source: Anal Chem. 2022 Mar 17;94(12):5029–40. doi: 10.1021/acs.analchem.1c04985 (PMC8968871; doi:10.1021/acs.analchem.1c04985)
Supplement: Supplementary file 1 — ac1c04985_si_001.pdf [file ac1c04985_si_001.pdf]

Supplemental Information for: Utilization of Machine Learning for the Differentiation of Positional NPS Isomers with Direct Analysis in Real Time Mass Spectrometry

Jennifer L. Bonetti,[*,†,‡] Saer Samanipour,[†] and Arian C. van Asten[†,¶]

†*Van 't Hoff Institute for Molecular Sciences, University of Amsterdam, P.O. Box 94157, Amsterdam 1090 GD, The Netherlands*

‡*Virginia Department of Forensic Science, Norfolk, VA, 23606 USA*

¶*Co van Ledden Hulsebosch Center (CLHC), Amsterdam Center for Forensic Science and Medicine, 1098 XH Amsterdam, The Netherlands*

E-mail: J.L.Bonetti@uva.nl

List of Figures

S1 Structures of isomers studied. S4
S2 Example DART 30/60/90V spectra S5
S3 Variable selection example S6
S4 Week-to-week example - FMA S11
S5 Accumulating dataset example - FMA S12
S6 Receiver Operator Characteristic Curves - FA S12
S7 Receiver Operator Characteristic Curves - MMC S13
S8 LDA Scores plots - FA S14
S9 LDA Scores plots - MMC S15
S10 Correlation Heat Map - FA S16
S11 Correlation Heat Map - FMA S17
S12 Correlation Heat Map - MMC S18
S13 Comparison of Classification Models - FA S19
S14 Comparison of Classification Models - FMA S20
S15 Comparison of Classification Models - MMC S21
S16 Comparison of Classification Models - FA - Same day S22
S17 Comparison of Classification Models - FMA - Same day S23
S18 Comparison of Classification Models - MMC - Same day S24
S19 Variable Importance - FA S25
S20 Variable Importance - FMA S26
S21 Variable Importance - MMC S27

List of Tables

S1 DART Method Parameters. S4

S2 QuickStrip™ Sample Locations. S5

S3 Final m/z bins retained for analysis. S7

S4 Normalization example - FMA S7

S5 ANOVA Results S7

S6 Mean abundance - FA S8

S7 Mean abundance - FMA S9

S8 Mean abundance - MMC S10

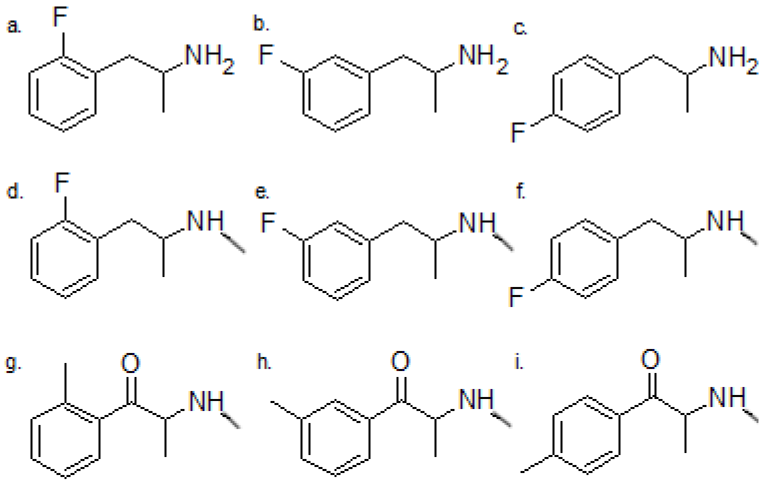

Figure S1: Structures of isomers studied. a-c: 2-, 3-, 4- fluoroamphetamine, respectively, d-f: 2-, 3-, 4- fluoromethamphetamine, respectively, and g-i: 2-, 3-, 4-, methylmethcathinone, respectively.

Table S1: DART Method Parameters.

Parameter	Setting
DART Temperature	350°C
DART Gas	Helium
Gas Flow Rate	3.5 L/min
Orifice 1 Voltage	20V, 30V, 60V, 90V switching at 0.25s
Ring Lens Voltage	15V
Orifice 2 Voltage	5V
Ion Guide Voltage	500V
m/z Scan Range	m/z 66 - m/z 600
Linear Rail Speed	0.3 mm/sec

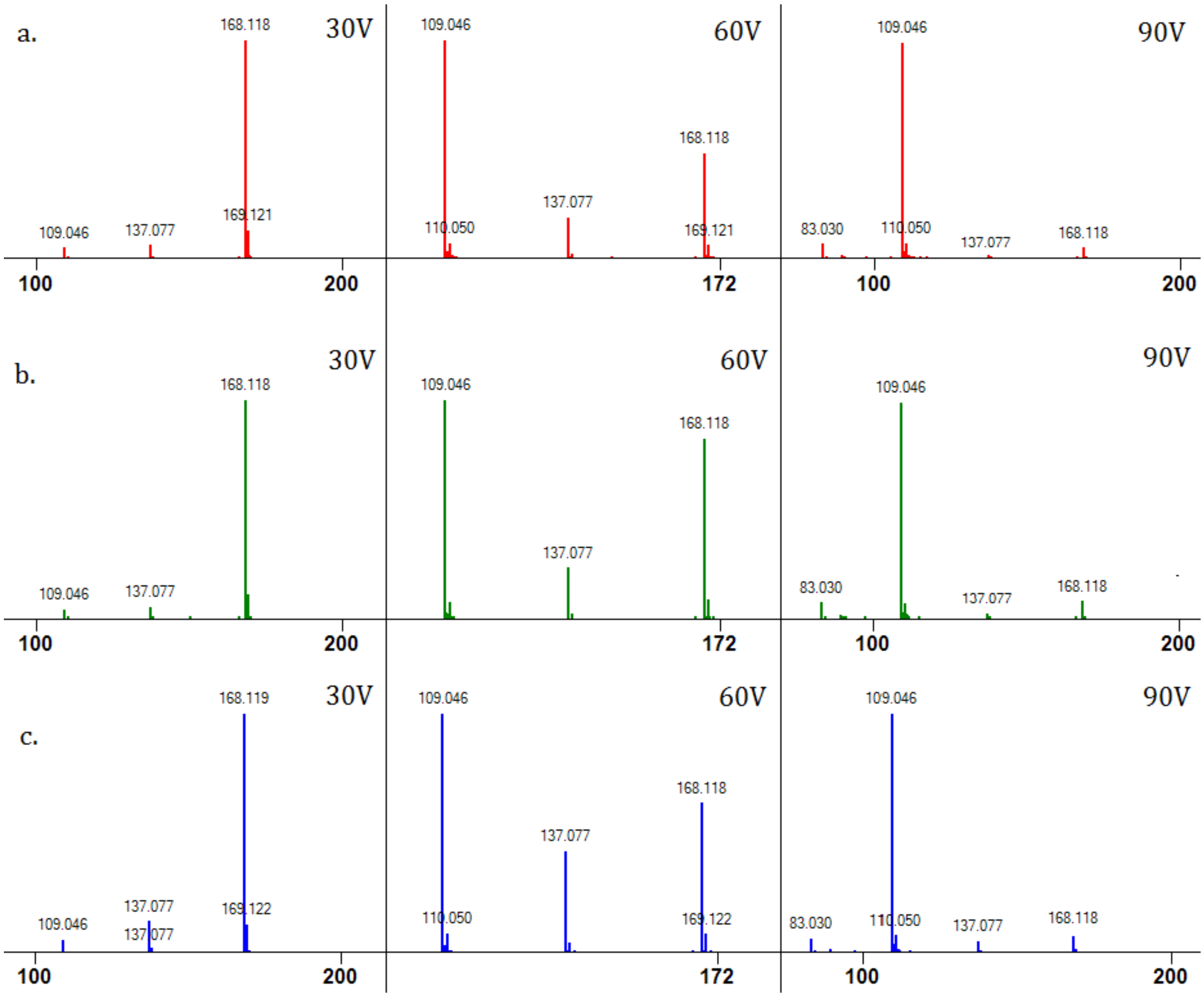

Figure S2: Example DART 30/60/90V spectra for FMA. a) 2-FMA, b) 3-FMA, and c)4-FMA

Table S2: QuickStrip™ Sample Locations.

Location(s)	Sample Type
1	PEG 600 calibrator solution
2	QA drug mix
3	Solvent blank
4-6	2-isomer
7-9	3-isomer
10-12	4-isomer

1. Initial dataset
    a. 288 samples x 3 voltages = 864 rows
    b. Mass range 66 - 170 in bins of 0.025 Da width = 4161 columns

2. Remove bins with all 0 signal
    a. 864 rows
    b. 380 columns

3. Identify highest abundance per sample. Identify which bins per sample have an abundance value above 0.3% of the highest abundance. For each bin, determine how many samples have reached the percent abundance threshold. Remove bins which have fewer than 50 samples which meet the threshold
    a. 864 rows
    b. 29 columns
        i. 83.050, 84.050, 88.025, 89.050, 109.050, 109.150, 109.425, 109.500, 109.575, 109.850, 110.050, 110.175, 110.550, 110.900, 111.275, 111.600, 111.625, 111.975, 112.325, 115.075, 137.100, 137.175, 138.100, 166.125, 168.125, 168.600, 168.675, 169.125, 169.550

4. Separate by voltage

30V	60V	90V
288 rows	288 rows	288 rows
29 columns	29 columns	29 columns

5. Remove bins whose mean abundance is less than 120 (instrumental centroiding threshold)

30V	60V	90V
288 rows	288 rows	288 rows
10 columns	24 columns	23 columns

6. Remove any remaining bins where over 2/3 of samples of 0 abundance

30V	60V	90V
288 rows	288 rows	288 rows
9 columns	20 columns	21 columns

7. Remove suspected fringe bins

30V	60V	90V
288 rows	288 rows	288 rows
7 columns	10 columns	13 columns

8. Remove suspected isotope bins

30V	60V	90V
288 rows	288 rows	288 rows
4 columns	6 columns	8 columns

9. Recombine voltages for final dataset
    a. 288 rows
    b. 18 columns (4 30V, 6 60V, 8 90V)

Figure S3: Walk-through example of m/z bin selection for 0.3% abundance threshold FMA dataset.

Table S3: Final m/z bins retained for analysis.

Isomer Type	Percentage Threshold	30V	60V	90V
FA	0.3	109.050, 119.100, 136.125, 137.100, 152.100, 154.125	83.050, 88.025, 91.075, 109.050 135.075, 136.125, 137.100, 152.100, 154.125	83.050, 89.050, 91.075, 109.050, 115.075, 135.075, 137.100, 152.100, 154.125
	10	109.050, 137.100, 154.125	83.050, 109.050, 137.100, 154.125	83.050, 109.050, 137.100, 154.125
FMA	0.3	109.050, 137.100 166.125, 168.125	83.050, 88.025, 109.050, 137.100, 166.125, 168.125	83.050, 89.050, 109.050, 112.325, 115.075, 137.100, 166.125, 168.125
	10	109.050, 137.100, 168.125	83.050, 109.050, 137.100, 168.125	83.050, 109.050, 137.100, 168.125
MMC	1	144.100, 145.100, 147.100, 160.125, 178.125	117.075, 119.100, 129.075, 130.075, 144.100, 145.100, 147.100, 158.100, 160.125, 178.125	91.072, 103.075, 104.075, 115.075, 117.075, 119.100, 128.075, 129.075, 130.075, 131.075, 144.100, 145.100, 147.100, 158.100, 160.125, 178.125
	10	144.100, 145.100, 160.125, 178.125	119.100, 144.100, 145.100, 160.125, 178.125	119.100, 144.100, 145.100, 160.125, 178.125

Table S4: Example normalization for one FMA sample 30V data (0.3% abundance threshold). m/z bins labeled as upper limit shown in Daltons

	109.050	137.100	166.125	168.125	Sum	Root Sum Square
Sample 1 Raw Abundance	3131.40	4250.89	463.70	75322.54	83169.54	75508.82
Sample 1 normalized by Ion Current	0.03766	0.05111	0.00558	0.90565	1	-
Sample 1 normalized by Vector Length	0.04148	0.05630	0.00614	0.99753	-	1

Table S5: ANOVA results showing average p-value ± standard deviation for each category. Number of m/z bins resulting in p-value > 0.05 is shown in parentheses.

Isomer	% Abundance	Normalization	Isomer Identity	Week	Volume
FA	0.3	Vector Length	$1.25 \times 10^{-3} \pm 6.07 \times 10^{-3}$ (0)	$3.69 \times 10^{-4} \pm 1.49 \times 10^{-3}$ (0)	$1.88 \times 10^{-1} \pm 2.36 \times 10^{-1}$ (13)
		Ion Current	$3.63 \times 10^{-2} \pm 1.78 \times 10^{-1}$ (1)	$3.34 \times 10^{-5} \pm 1.09 \times 10^{-4}$ (0)	$1.81 \times 10^{-1} \pm 2.49 \times 10^{-1}$ (13)
	10	Vector Length	$2.76 \times 10^{-5} \pm 7.59 \times 10^{-5}$ (0)	$6.79 \times 10^{-4} \pm 2.11 \times 10^{-3}$ (0)	$1.89 \times 10^{-1} \pm 1.83 \times 10^{-1}$ (7)
		Ion Current	$8.32 \times 10^{-2} \pm 2.76 \times 10^{-1}$ (1)	$4.61 \times 10^{-5} \pm 1.36 \times 10^{-4}$ (0)	$1.68 \times 10^{-1} \pm 1.71 \times 10^{-1}$ (7)
FMA	0.3	Vector Length	$6.13 \times 10^{-4} \pm 1.23 \times 10^{-3}$ (0)	$8.65 \times 10^{-6} \pm 3.57 \times 10^{-5}$ (0)	$2.20 \times 10^{-1} \pm 2.81 \times 10^{-1}$ (11)
		Ion Current	$5.34 \times 10^{-3} \pm 1.19 \times 10^{-2}$ (0)	$1.31 \times 10^{-5} \pm 5.54 \times 10^{-5}$ (0)	$2.20 \times 10^{-1} \pm 3.13 \times 10^{-1}$ (9)
	10	Vector Length	$3.38 \times 10^{-4} \pm 9.74 \times 10^{-4}$ (0)	$2.51 \times 10^{-16} \pm 8.32 \times 10^{-16}$ (0)	$2.27 \times 10^{-1} \pm 3.10 \times 10^{-1}$ (7)
		Ion Current	$3.13 \times 10^{-3} \pm 8.11 \times 10^{-3}$ (0)	$1.99 \times 10^{-19} \pm 6.59 \times 10^{-19}$ (0)	$2.53 \times 10^{-1} \pm 3.61 \times 10^{-1}$ (6)
MMC	1	Vector Length	$3.51 \times 10^{-2} \pm 1.62 \times 10^{-1}$ (3)	$1.52 \times 10^{-5} \pm 7.83 \times 10^{-5}$ (0)	$1.80 \times 10^{-1} \pm 2.71 \times 10^{-1}$ (14)
		Ion Current	$2.42 \times 10^{-2} \pm 8.10 \times 10^{-2}$ (3)	$7.95 \times 10^{-6} \pm 4.35 \times 10^{-5}$ (0)	$1.77 \times 10^{-1} \pm 2.81 \times 10^{-1}$ (14)
	10	Vector Length	$8.02 \times 10^{-3} \pm 3.00 \times 10^{-2}$ (1)	$2.98 \times 10^{-7} \pm 1.05 \times 10^{-6}$ (0)	$1.89 \times 10^{-1} \pm 3.00 \times 10^{-1}$ (6)
		Ion Current	$4.01 \times 10^{-2} \pm 1.11 \times 10^{-1}$ (2)	$2.51 \times 10^{-7} \pm 9.30 \times 10^{-7}$ (0)	$1.81 \times 10^{-1} \pm 2.95 \times 10^{-1}$ (6)

Table S6: Mean abundance values and associated standard deviation for FA using a 0.3% abundance threshold dataset with ion current normalization.

	2-FA		3-FA		4-FA	
m/z / Voltage	Mean	s.d.	Mean	s.d.	Mean	s.d.
109.050 30V	$9.10x10^{-2}$	$1.79x10^{-2}$	$9.03x10^{-2}$	$1.53x10^{-2}$	$9.13x10^{-2}$	$1.63x10^{-2}$
119.100 30V	$4.58x10^{-3}$	$2.14x10^{-3}$	$4.15x10^{-3}$	$2.34x10^{-3}$	$3.38x10^{-3}$	$3.34x10^{-3}$
136.125 30V	$1.61x10^{-2}$	$3.45x10^{-3}$	$1.65x10^{-2}$	$5.94x10^{-3}$	$5.48x10^{-3}$	$2.93x10^{-3}$
137.100 30V	$1.86x10^{-1}$	$2.81x10^{-2}$	$1.96x10^{-1}$	$2.40x10^{-2}$	$3.47x10^{-1}$	$3.72x10^{-2}$
152.100 30V	$5.33x10^{-3}$	$2.23x10^{-3}$	$5.68x10^{-3}$	$2.20x10^{-3}$	$6.61x10^{-3}$	$2.46x10^{-3}$
154.125 30V	$6.97x10^{-1}$	$4.17x10^{-2}$	$6.88x10^{-1}$	$3.27x10^{-2}$	$5.46x10^{-1}$	$4.80x10^{-2}$
83.050 60V	$2.38x10^{-3}$	$1.04x10^{-3}$	$2.89x10^{-3}$	$1.26x10^{-3}$	$2.42x10^{-3}$	$1.08x10^{-3}$
88.025 60V	$7.20x10^{-4}$	$5.94x10^{-4}$	$2.99x10^{-3}$	$1.34x10^{-3}$	$1.10x10^{-3}$	$6.09x10^{-4}$
91.075 60V	$6.17x10^{-3}$	$4.89x10^{-3}$	$4.28x10^{-3}$	$5.02x10^{-3}$	$8.33x10^{-4}$	$6.56x10^{-4}$
109.050 60V	$6.35x10^{-1}$	$7.96x10^{-2}$	$5.95x10^{-1}$	$8.16x10^{-2}$	$5.94x10^{-1}$	$8.97x10^{-2}$
135.075 60V	$7.79x10^{-4}$	$5.68x10^{-4}$	$1.05x10^{-3}$	$5.42x10^{-4}$	$1.44x10^{-3}$	$4.82x10^{-4}$
136.125 60V	$4.22x10^{-3}$	$1.71x10^{-3}$	$4.45x10^{-3}$	$2.05x10^{-3}$	$1.23x10^{-3}$	$6.87x10^{-4}$
137.100 60V	$1.34x10^{-1}$	$1.73x10^{-2}$	$1.33x10^{-1}$	$1.52x10^{-2}$	$2.00x10^{-1}$	$2.85x10^{-2}$
152.100 60V	$3.52x10^{-3}$	$1.08x10^{-3}$	$4.24x10^{-3}$	$1.14x10^{-3}$	$4.75x10^{-3}$	$1.15x10^{-3}$
154.125 60V	$2.14x10^{-1}$	$6.54x10^{-2}$	$2.52x10^{-1}$	$6.95x10^{-2}$	$1.94x10^{-1}$	$6.53x10^{-2}$
83.050 90V	$7.44x10^{-2}$	$3.07x10^{-2}$	$8.34x10^{-2}$	$3.50x10^{-2}$	$6.97x10^{-2}$	$2.94x10^{-2}$
89.050 90V	$1.15x10^{-2}$	$2.90x10^{-3}$	$1.28x10^{-2}$	$3.50x10^{-3}$	$1.08x10^{-2}$	$3.33x10^{-3}$
91.075 90V	$7.55x10^{-3}$	$6.29x10^{-3}$	$5.39x10^{-3}$	$6.04x10^{-3}$	$9.75x10^{-4}$	$8.55x10^{-4}$
109.050 90V	$8.19x10^{-1}$	$2.79x10^{-2}$	$8.05x10^{-1}$	$3.14x10^{-2}$	$8.19x10^{-1}$	$3.25x10^{-2}$
115.075 90V	$3.75x10^{-3}$	$7.63x10^{-4}$	$2.42x10^{-3}$	$5.86x10^{-4}$	$2.80x10^{-3}$	$7.69x10^{-4}$
135.075 90V	$9.10x10^{-4}$	$5.83x10^{-4}$	$1.73x10^{-3}$	$5.64x10^{-4}$	$2.82x10^{-3}$	$6.49x10^{-4}$
137.100 90V	$3.51x10^{-2}$	$1.06x10^{-2}$	$3.89x10^{-2}$	$1.29x10^{-2}$	$5.82x10^{-2}$	$1.96x10^{-2}$
152.100 90V	$1.31x10^{-3}$	$5.96x10^{-4}$	$2.35x10^{-3}$	$9.48x10^{-4}$	$2.75x10^{-3}$	$1.01x10^{-3}$
154.125 90V	$4.68x10^{-2}$	$1.73x10^{-2}$	$4.77x10^{-2}$	$2.30x10^{-2}$	$3.28x10^{-2}$	$1.70x10^{-2}$

Table S7: Mean abundance values and associated standard deviation for FMA using a 0.3% abundance threshold dataset with ion current normalization

	2-FMA		3-FMA		4-FMA	
m/z / Voltage	Mean	s.d.	Mean	s.d.	Mean	s.d.
109.050 30V	4.05×10^{-2}	8.03×10^{-3}	3.57×10^{-2}	7.85×10^{-3}	4.32×10^{-2}	8.93×10^{-3}
137.100 30V	4.82×10^{-2}	1.07×10^{-2}	4.39×10^{-2}	1.10×10^{-2}	1.26×10^{-1}	2.78×10^{-2}
166.125 30V	2.81×10^{-3}	1.24×10^{-3}	3.21×10^{-3}	1.24×10^{-3}	3.09×10^{-3}	1.41×10^{-3}
168.125 30V	9.08×10^{-1}	1.83×10^{-2}	9.17×10^{-1}	1.81×10^{-2}	8.27×10^{-1}	3.58×10^{-2}
83.050 60V	1.16×10^{-3}	5.04×10^{-4}	1.33×10^{-3}	5.72×10^{-4}	1.31×10^{-3}	5.75×10^{-4}
88.025 60V	1.49×10^{-3}	1.04×10^{-3}	7.31×10^{-4}	3.75×10^{-4}	1.48×10^{-3}	1.08×10^{-3}
109.050 60V	5.44×10^{-1}	7.73×10^{-2}	4.81×10^{-1}	9.94×10^{-2}	5.16×10^{-1}	9.50×10^{-2}
137.100 60V	1.10×10^{-1}	6.38×10^{-3}	1.05×10^{-1}	8.25×10^{-3}	2.06×10^{-1}	1.60×10^{-2}
166.125 60V	2.51×10^{-3}	8.18×10^{-4}	3.01×10^{-3}	9.48×10^{-4}	2.23×10^{-3}	8.30×10^{-4}
168.125 60V	3.41×10^{-1}	7.64×10^{-2}	4.09×10^{-1}	1.03×10^{-1}	2.73×10^{-1}	8.22×10^{-2}
83.050 90V	6.26×10^{-2}	2.52×10^{-2}	6.89×10^{-2}	3.02×10^{-2}	6.37×10^{-2}	2.86×10^{-2}
89.050 90V	9.43×10^{-3}	2.41×10^{-3}	1.01×10^{-2}	3.19×10^{-3}	9.26×10^{-3}	2.81×10^{-3}
109.050 90V	8.34×10^{-1}	2.86×10^{-2}	8.14×10^{-1}	3.65×10^{-2}	8.39×10^{-1}	2.62×10^{-2}
112.325 90V	1.25×10^{-3}	1.33×10^{-3}	1.04×10^{-3}	9.55×10^{-4}	1.71×10^{-3}	1.69×10^{-3}
115.075 90V	3.13×10^{-3}	6.55×10^{-4}	1.28×10^{-3}	4.87×10^{-4}	1.90×10^{-3}	4.67×10^{-4}
137.100 90V	1.60×10^{-2}	5.26×10^{-3}	1.91×10^{-2}	6.57×10^{-3}	3.38×10^{-2}	1.37×10^{-2}
166.125 90V	1.04×10^{-3}	5.54×10^{-4}	1.26×10^{-3}	4.97×10^{-4}	6.17×10^{-4}	4.36×10^{-4}
168.125 90V	7.28×10^{-2}	3.19×10^{-2}	8.41×10^{-2}	4.43×10^{-2}	4.96×10^{-2}	2.45×10^{-2}

Table S8: Mean abundance values and associated standard deviation for MMC using a 1% abundance threshold dataset with ion current normalization

	2-MMC		3-MMC		4-MMC	
m/z / Voltage	Mean	s.d.	Mean	s.d.	Mean	s.d.
144.100 30V	$3.27x10^{-3}$	$2.68x10^{-3}$	$3.60x10^{-3}$	$2.67x10^{-3}$	$4.47x10^{-3}$	$3.54x10^{-3}$
145.100 30V	$9.23x10^{-3}$	$4.24x10^{-3}$	$1.06x10^{-2}$	$5.44x10^{-3}$	$1.23x10^{-2}$	$7.16x10^{-3}$
147.100 30V	$5.28x10^{-3}$	$1.42x10^{-3}$	$4.58x10^{-3}$	$1.26x10^{-3}$	$6.04x10^{-3}$	$1.67x10^{-3}$
160.125 30V	$1.08x10^{-1}$	$2.62x10^{-2}$	$7.60x10^{-2}$	$1.56x10^{-2}$	$9.32x10^{-2}$	$2.11x10^{-2}$
178.125 30V	$8.74x10^{-1}$	$2.87x10^{-2}$	$9.05x10^{-1}$	$1.81x10^{-2}$	$8.84x10^{-1}$	$2.47x10^{-2}$
117.075 60V	$1.33x10^{-3}$	$5.22x10^{-4}$	$1.04x10^{-3}$	$4.38x10^{-4}$	$1.10x10^{-3}$	$5.07x10^{-4}$
119.100 60V	$2.56x10^{-2}$	$6.17x10^{-3}$	$2.66x10^{-2}$	$5.61x10^{-3}$	$2.68x10^{-2}$	$7.22x10^{-3}$
129.075 60V	$2.97x10^{-3}$	$6.31x10^{-4}$	$1.52x10^{-3}$	$3.43x10^{-4}$	$1.45x10^{-3}$	$3.93x10^{-4}$
130.075 60V	$1.58x10^{-3}$	$4.91x10^{-4}$	$8.99x10^{-4}$	$2.83x10^{-4}$	$9.92x10^{-4}$	$4.01x10^{-4}$
144.100 60V	$9.45x10^{-3}$	$3.07x10^{-3}$	$6.18x10^{-3}$	$1.86x10^{-3}$	$6.35x10^{-3}$	$2.00x10^{-3}$
145.100 60V	$1.95x10^{-1}$	$4.10x10^{-2}$	$1.48x10^{-1}$	$2.92x10^{-2}$	$1.50x10^{-1}$	$3.15x10^{-2}$
147.100 60V	$3.01x10^{-2}$	$1.84x10^{-3}$	$3.55x10^{-2}$	$1.74x10^{-3}$	$3.71x10^{-2}$	$1.73x10^{-3}$
158.100 60V	$1.33x10^{-3}$	$4.04x10^{-4}$	$5.78x10^{-4}$	$2.30x10^{-4}$	$5.98x10^{-4}$	$4.46x10^{-4}$
160.125 60V	$5.64x10^{-1}$	$2.76x10^{-2}$	$5.45x10^{-1}$	$1.76x10^{-2}$	$5.86x10^{-1}$	$1.75x10^{-2}$
178.125 60V	$1.69x10^{-1}$	$6.91x10^{-2}$	$2.34x10^{-1}$	$5.15x10^{-2}$	$1.89x10^{-1}$	$5.10x10^{-2}$
91.075 90V	$2.22x10^{-2}$	$8.02x10^{-3}$	$2.40x10^{-2}$	$8.39x10^{-3}$	$2.18x10^{-2}$	$1.02x10^{-2}$
103.075 90V	$5.64x10^{-3}$	$9.55x10^{-4}$	$5.49x10^{-3}$	$8.43x10^{-4}$	$5.58x10^{-3}$	$8.86x10^{-4}$
104.075 90V	$4.43x10^{-3}$	$6.85x10^{-4}$	$4.55x10^{-3}$	$6.45x10^{-4}$	$4.75x10^{-3}$	$5.88x10^{-4}$
115.075 90V	$4.01x10^{-3}$	$9.53x10^{-4}$	$3.63x10^{-3}$	$7.69x10^{-4}$	$3.61x10^{-3}$	$8.55x10^{-4}$
117.075 90V	$1.37x10^{-2}$	$1.23x10^{-3}$	$1.55x10^{-2}$	$1.25x10^{-3}$	$1.53x10^{-2}$	$1.75x10^{-3}$
119.100 90V	$3.53x10^{-2}$	$4.42x10^{-3}$	$4.92x10^{-2}$	$3.38x10^{-3}$	$4.67x10^{-2}$	$3.16x10^{-3}$
128.075 90V	$4.79x10^{-3}$	$6.36x10^{-4}$	$2.52x10^{-3}$	$3.26x10^{-4}$	$2.33x10^{-3}$	$4.36x10^{-4}$
129.075 90V	$5.47x10^{-3}$	$3.57x10^{-4}$	$3.40x10^{-3}$	$2.15x10^{-4}$	$2.92x10^{-3}$	$3.37x10^{-4}$
130.075 90V	$2.77x10^{-2}$	$3.81x10^{-3}$	$2.23x10^{-2}$	$2.93x10^{-3}$	$2.21x10^{-2}$	$3.20x10^{-3}$
131.075 90V	$5.63x10^{-3}$	$7.79x10^{-4}$	$4.26x10^{-3}$	$7.10x10^{-4}$	$3.85x10^{-3}$	$8.64x10^{-4}$
144.100 90V	$2.73x10^{-1}$	$4.09x10^{-2}$	$2.30x10^{-1}$	$3.17x10^{-2}$	$2.34x10^{-1}$	$3.53x10^{-2}$
145.100 90V	$4.45x10^{-1}$	$1.62x10^{-2}$	$4.49x10^{-1}$	$1.10x10^{-2}$	$4.49x10^{-1}$	$1.45x10^{-2}$
147.100 90V	$6.79x10^{-3}$	$1.77x10^{-3}$	$8.19x10^{-3}$	$1.67x10^{-3}$	$7.79x10^{-3}$	$2.23x10^{-3}$
158.100 90V	$4.66x10^{-3}$	$5.03x10^{-4}$	$3.69x10^{-3}$	$4.16x10^{-4}$	$3.45x10^{-3}$	$6.01x10^{-4}$
160.125 90V	$1.31x10^{-1}$	$3.37x10^{-2}$	$1.54x10^{-1}$	$2.95x10^{-2}$	$1.59x10^{-1}$	$3.81x10^{-2}$
178.125 90V	$9.80x10^{-3}$	$7.74x10^{-3}$	$2.10x10^{-2}$	$1.20x10^{-2}$	$1.83x10^{-2}$	$1.60x10^{-2}$

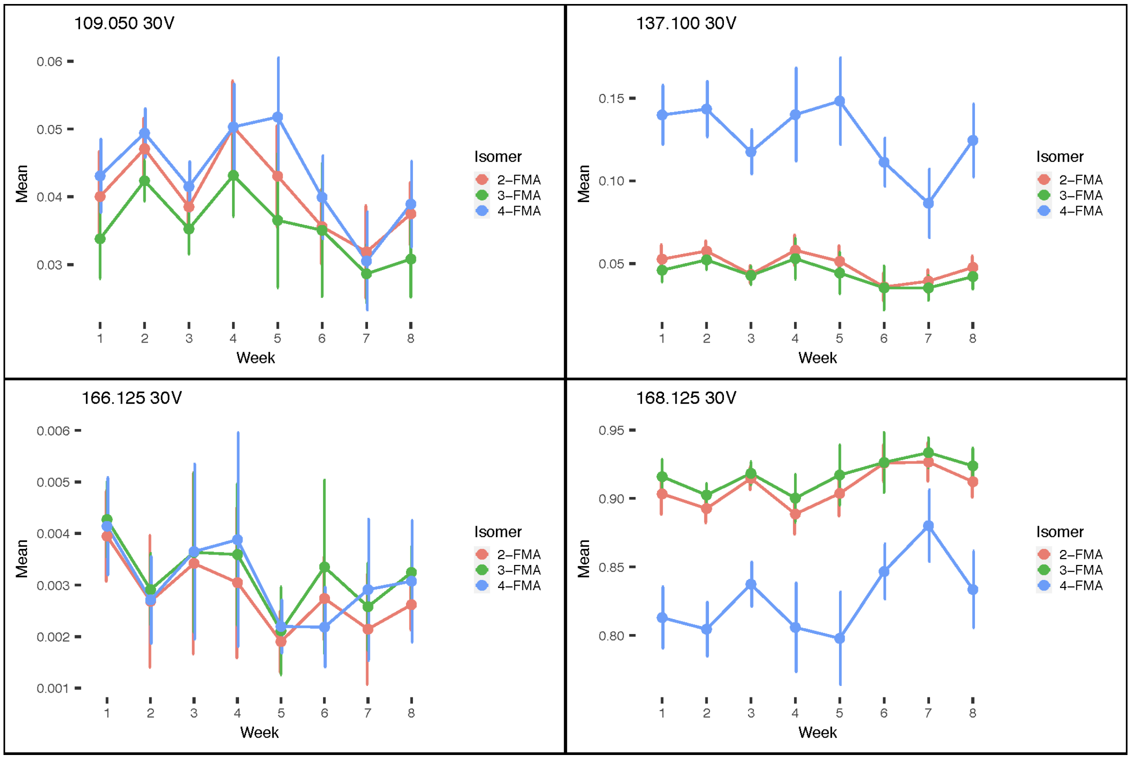

Figure S4: FMA 30V week-to-week data using ion current normalization and 0.3% abundance threshold.

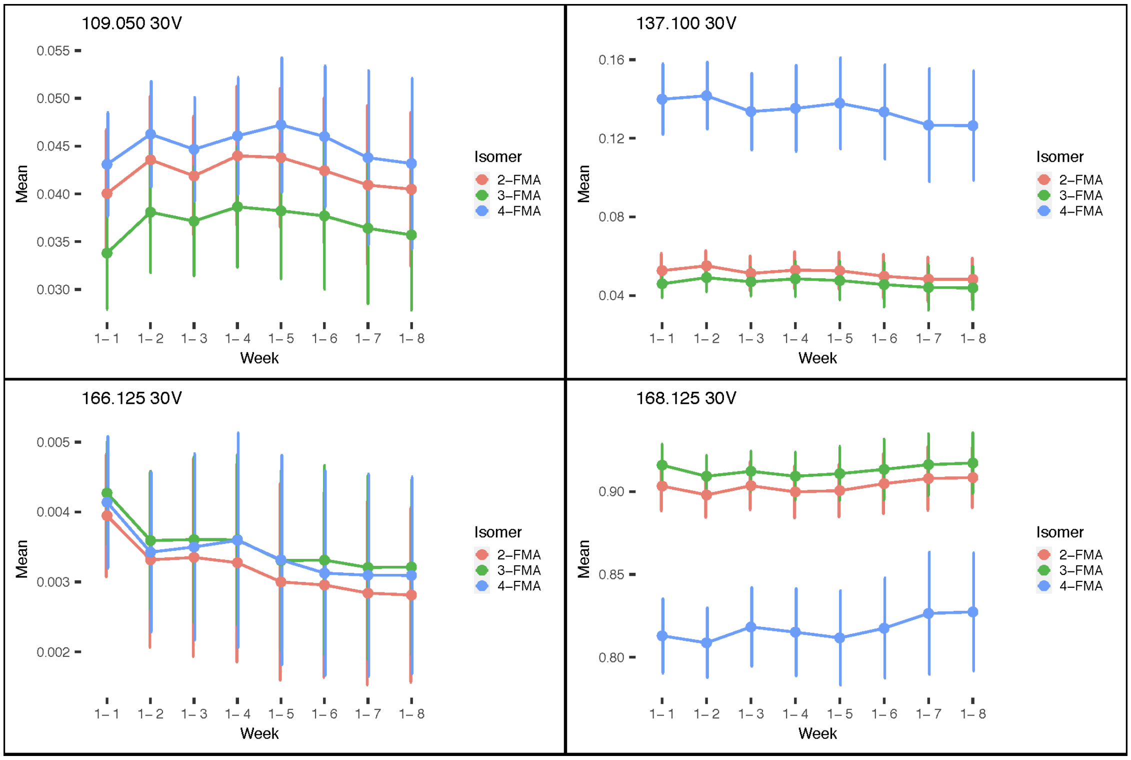

Figure S5: FMA 30V accumulating dataset over time using ion current normalization and 0.3% abundance threshold.

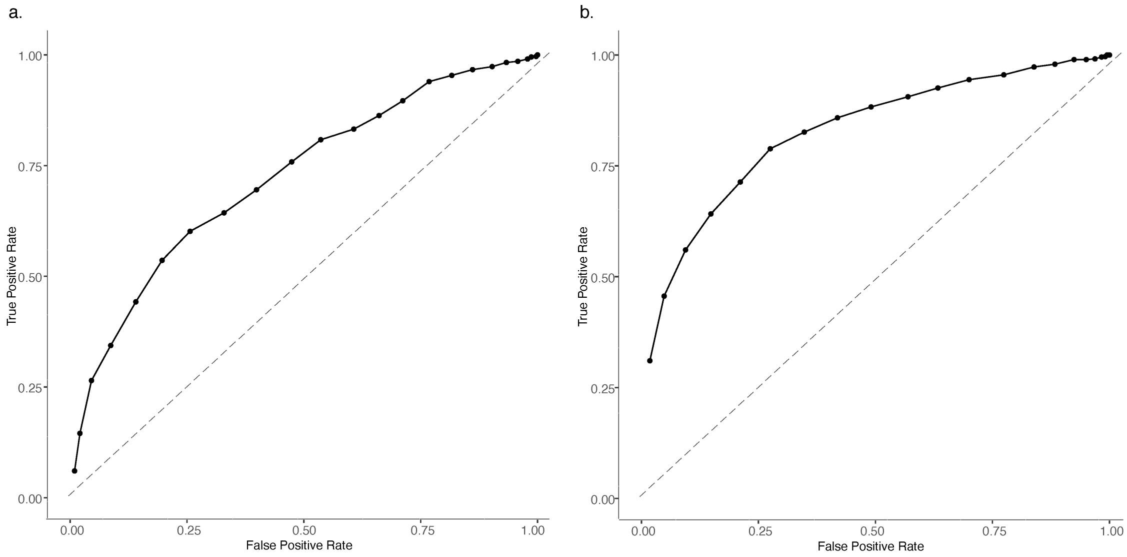

Figure S6: Receiver Operator Characteristic Curves for average of twenty replicates of Welch test analysis of FA isomers using a 0.3% abundance threshold. a) full dataset (AUC = 0.7452) and b) same day restricted dataset (AUC = 0.8460). Dotted line approximating a random classifier of AUC = 0.5 is shown.

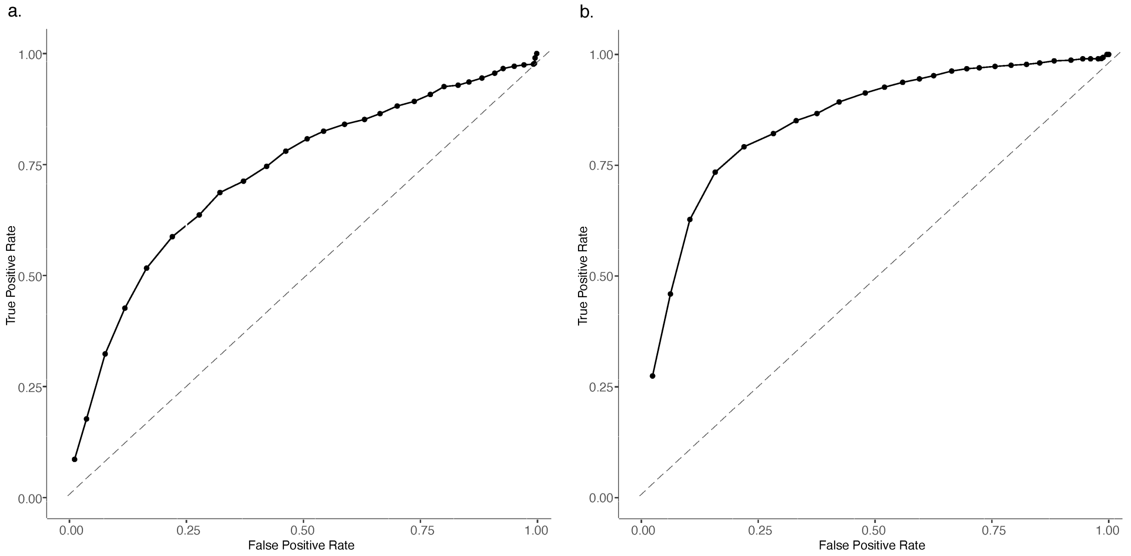

Figure S7: Receiver Operator Characteristic Curves for average of twenty replicates of Welch test analysis of MMC isomers using a 1% abundance threshold. a) full dataset (AUC = 0.7452) and b) same day restricted dataset (AUC = 0.8679). Dotted line approximating a random classifier of AUC = 0.5 is shown.

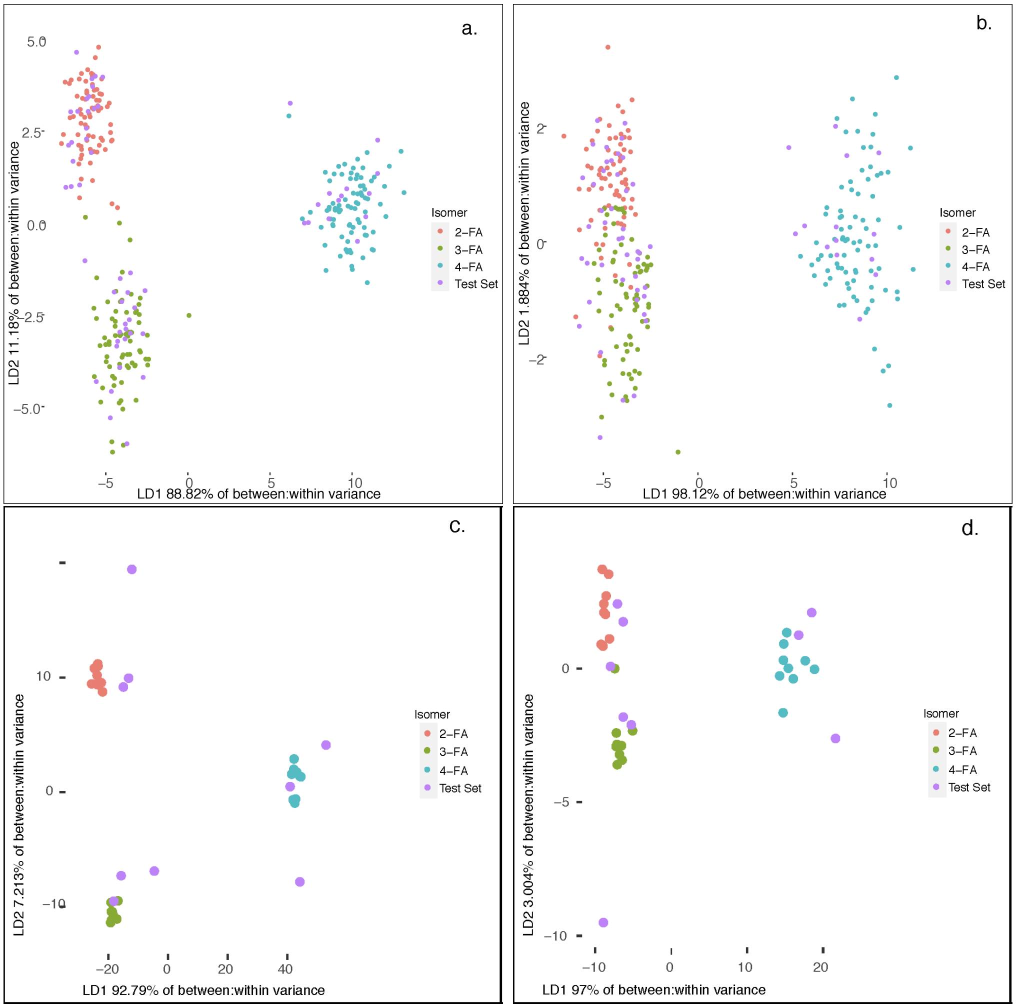

Figure S8: Linear Discriminant Analysis Scores plots for training vs. test sets of FA dataset using ion current normalization for: a) Full dataset 0.3% threshold, b) Full data set 10% threshold, c) Same day analysis (first week, first card as test set) 0.3% threshold, d) Same day analysis (first week, first card as test set) 10% threshold

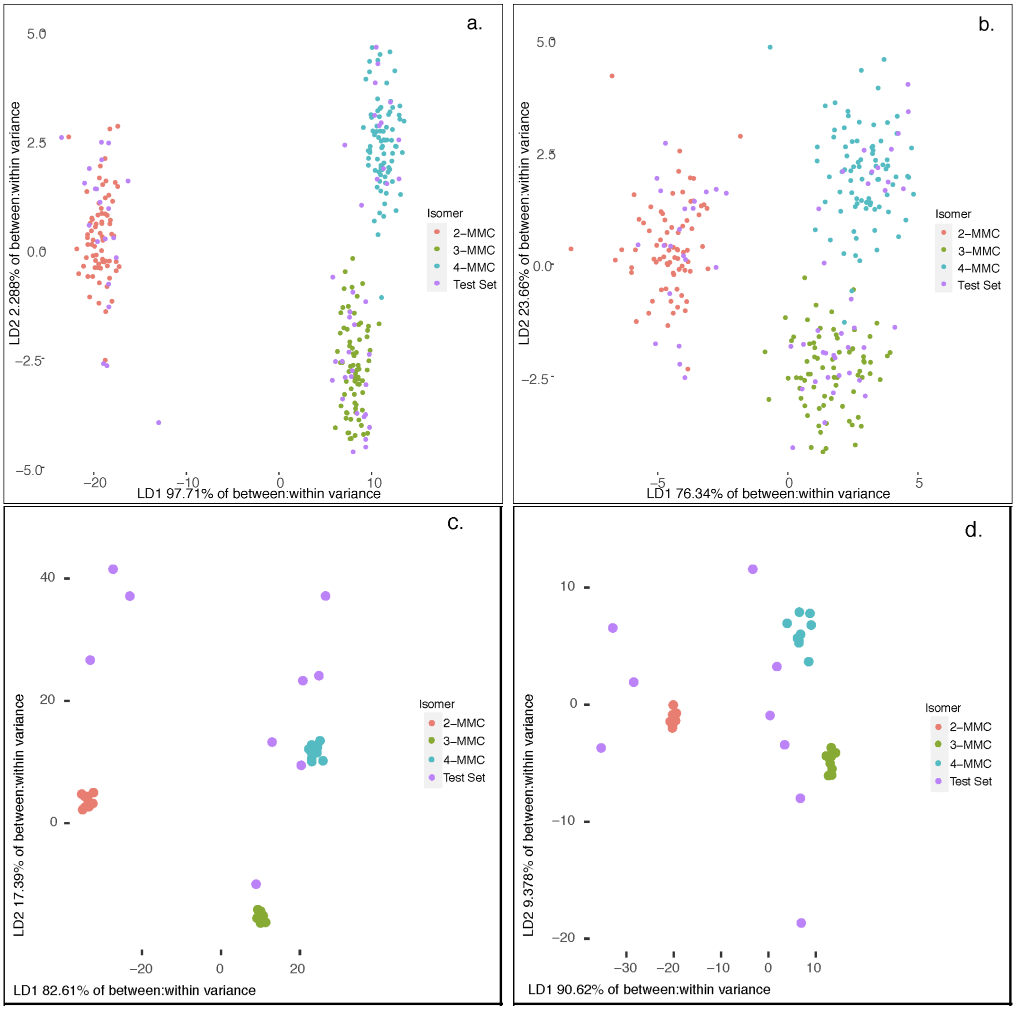

Figure S9: Linear Discriminant Analysis Scores plots for training vs. test sets of MMC dataset using ion current normalization for: a) Full dataset 1% threshold, b) Full data set 10% threshold, c) Same day analysis (first week, first card as test set) 1% threshold, d) Same day analysis (first week, first card as test set) 10% threshold

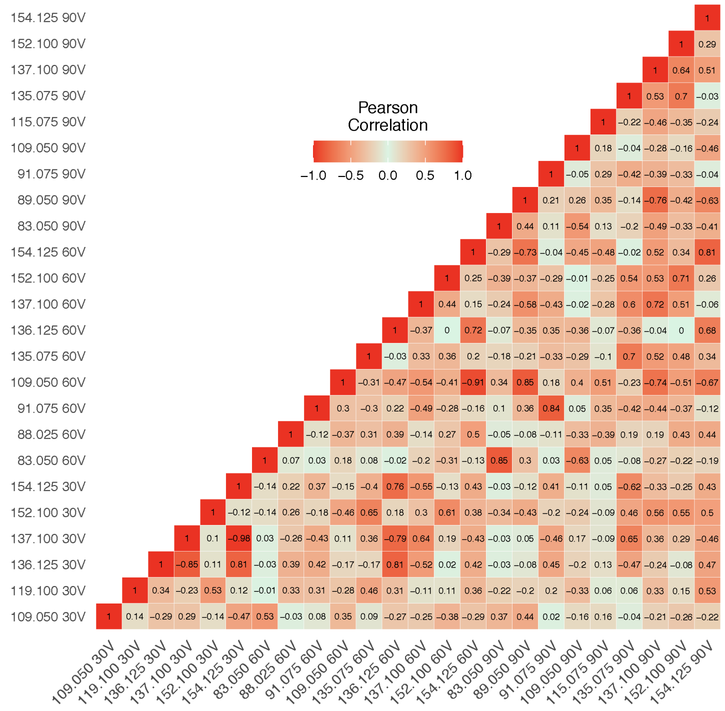

Figure S10: Correlation Heat Map for full FA data set with 0.3% abundance threshold using ion current normalization

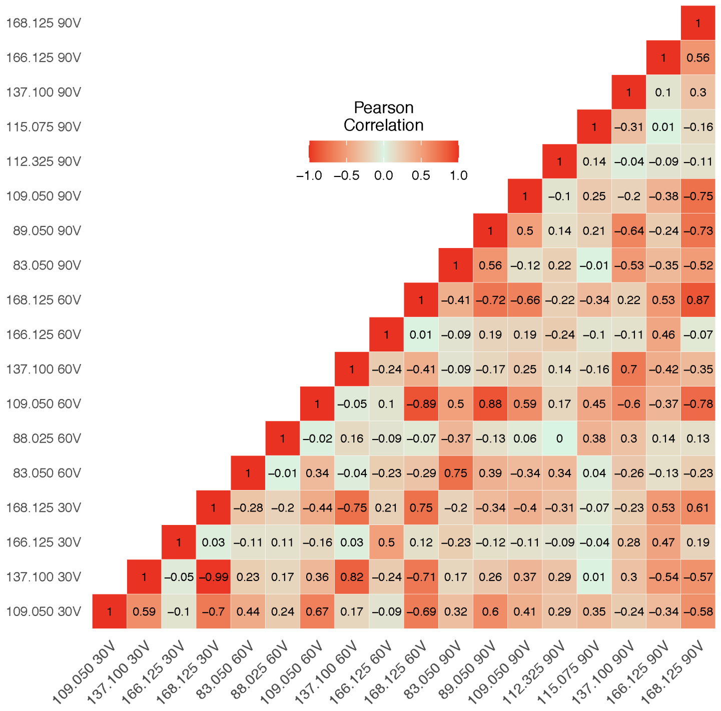

Figure S11: Correlation Heat Map for full FMA data set with 0.3% abundance threshold using ion current normalization

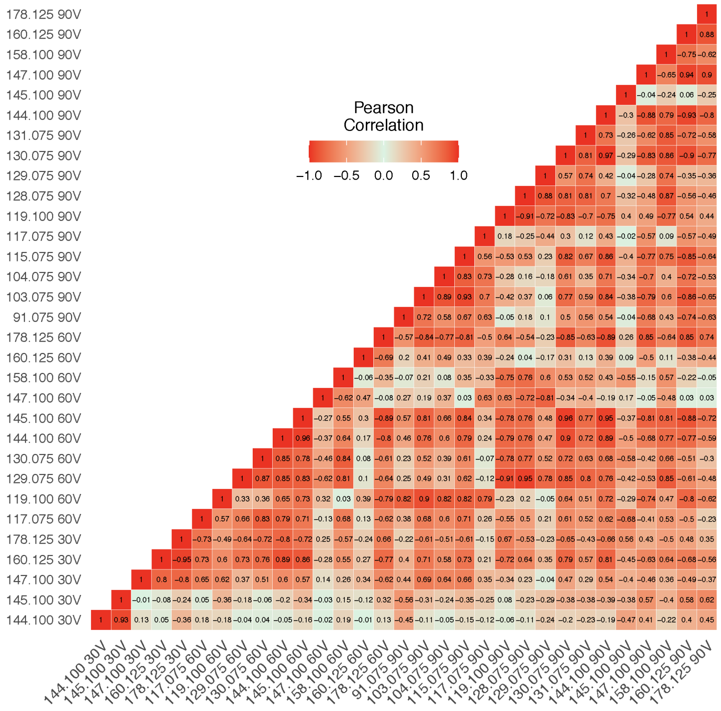

Figure S12: Correlation Heat Map for full MMC data set with 1% abundance threshold using ion current normalization

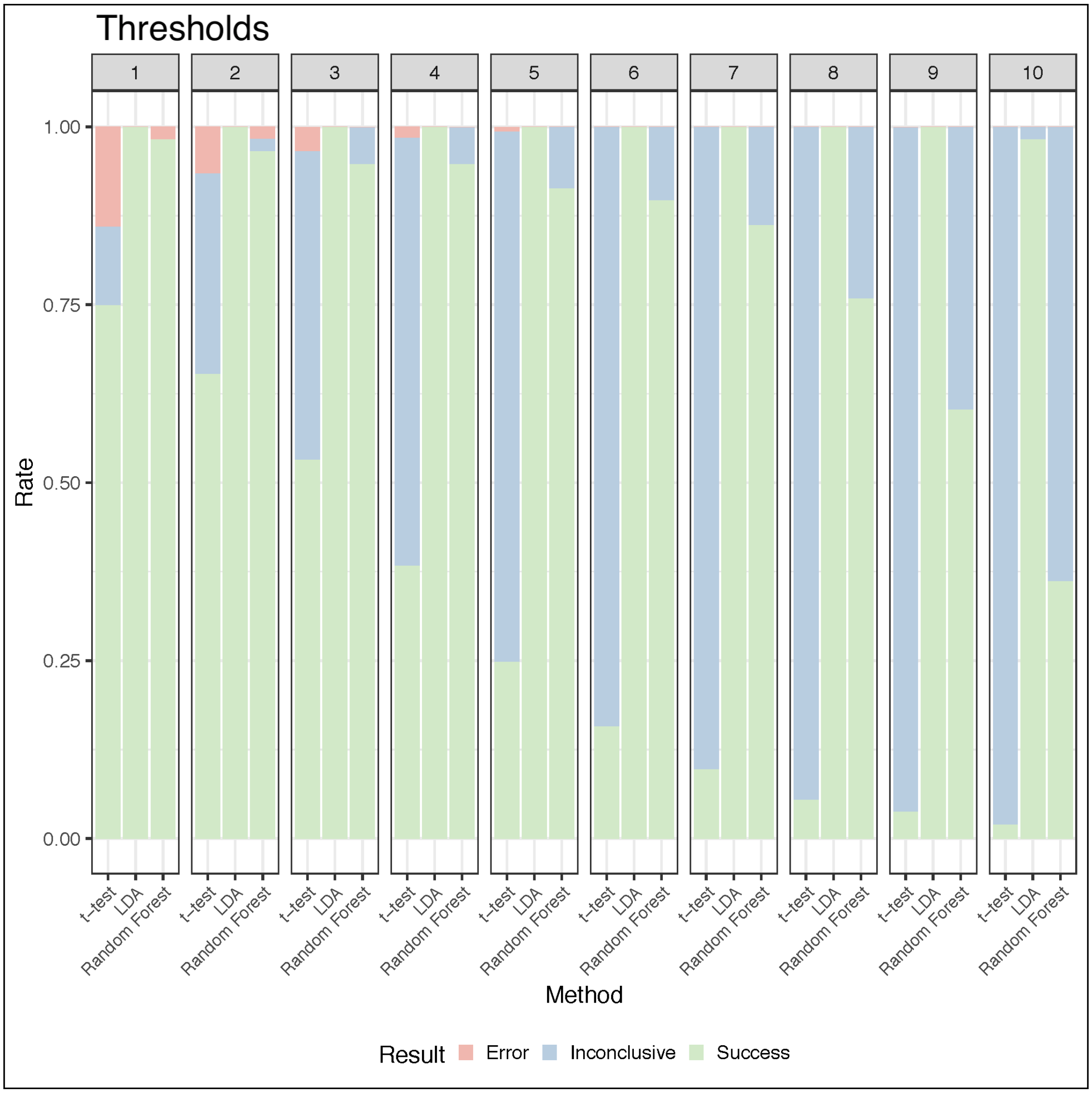

Figure S13: Comparison of success, inconclusive, and error rates for the three methods based on increasing thresholds for FA isomers. For t-test analysis, thresholds are the difference in the number of indistinguishable bins between the two most likely isomers. For LDA, the thresholds are the posterior probability of the classification, starting at 0.50 and increasing to 0.95, step-wise by 0.05. For Random Forest, the thresholds are the proportion of decision trees pointing to the classification, starting at 0.50 and increasing to 0.95, step-wise by 0.05. For LDA and Random Forest, ion current normalization was performed while the t-test used randomly assigned normalization per replicate.

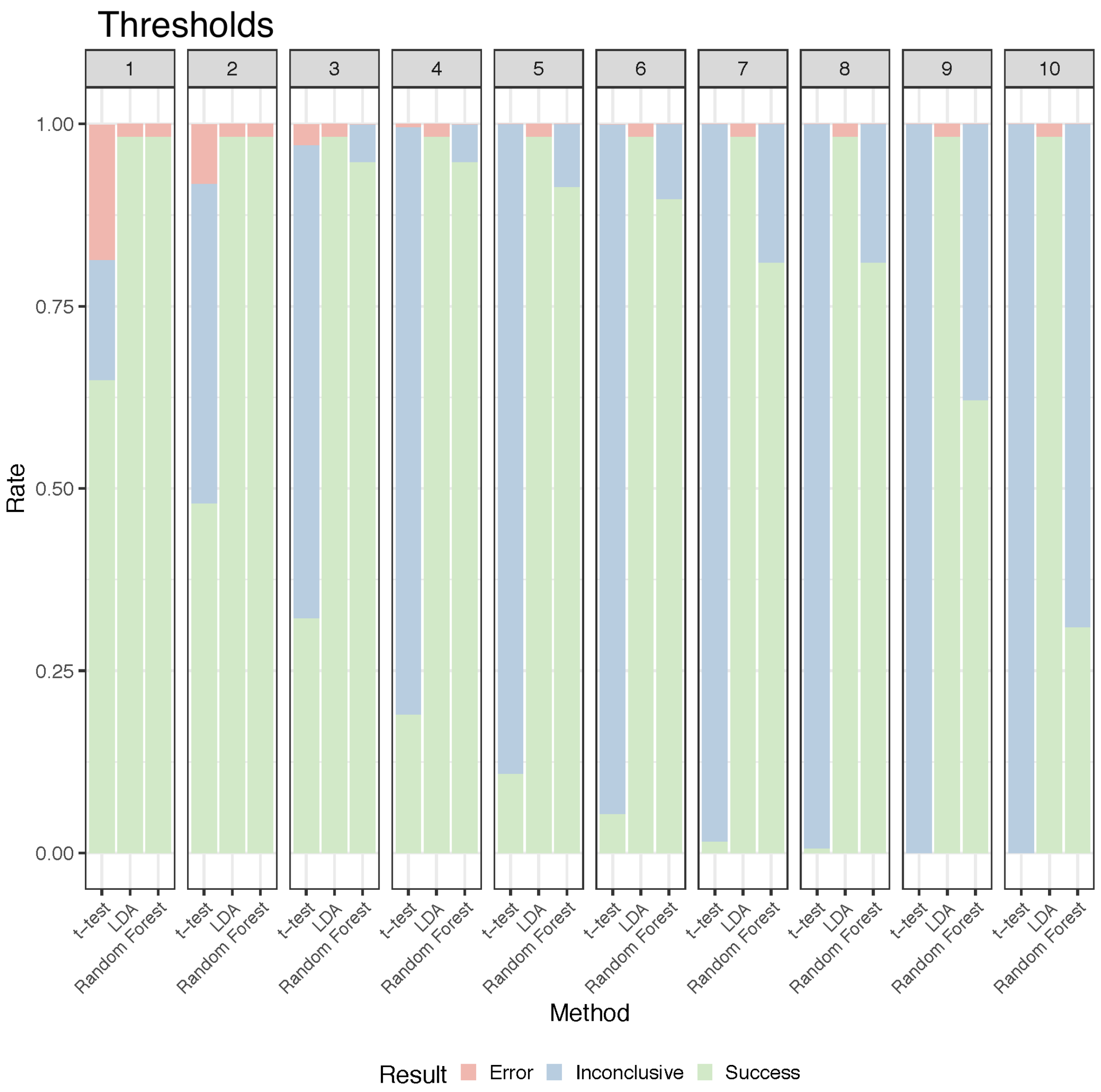

Figure S14: Comparison of success, inconclusive, and error rates for the three methods based on increasing thresholds for FMA isomers. For t-test analysis, thresholds are the difference in the number of indistinguishable bins between the two most likely isomers. For LDA, the thresholds are the posterior probability of the classification, starting at 0.50 and increasing to 0.95, step-wise by 0.05. For Random Forest, the thresholds are the proportion of decision trees pointing to the classification, starting at 0.50 and increasing to 0.95, step-wise by 0.05. For LDA and Random Forest, ion current normalization was performed while the t-test used randomly assigned normalization per replicate.

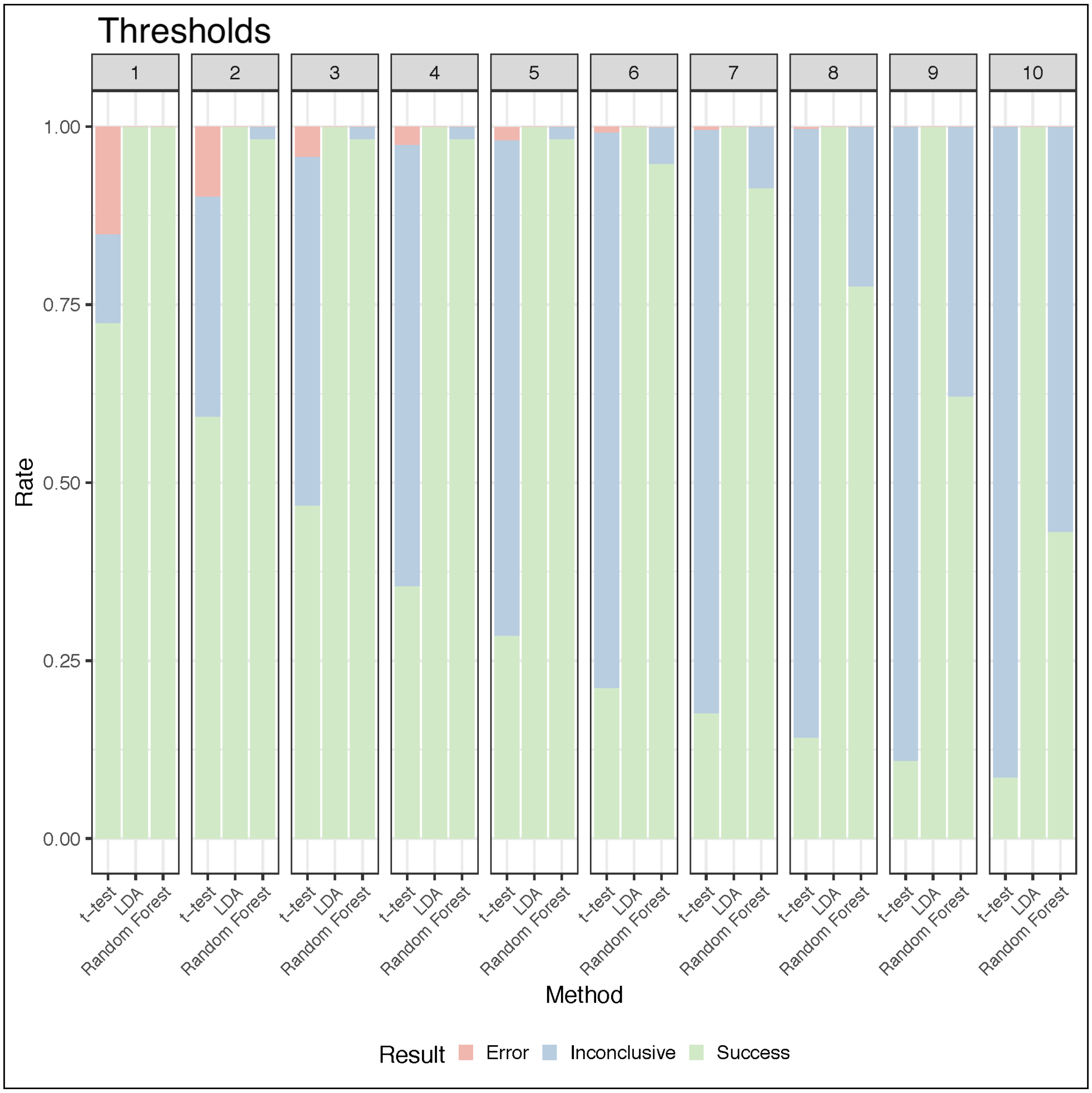

Figure S15: Comparison of success, inconclusive, and error rates for the three methods based on increasing thresholds for MMC isomers. For t-test analysis, thresholds are the difference in the number of indistinguishable bins between the two most likely isomers. For LDA, the thresholds are the posterior probability of the classification, starting at 0.50 and increasing to 0.95, step-wise by 0.05. For Random Forest, the thresholds are the proportion of decision trees pointing to the classification, starting at 0.50 and increasing to 0.95, step-wise by 0.05. For LDA and Random Forest, ion current normalization was performed while the t-test used randomly assigned normalization per replicate.

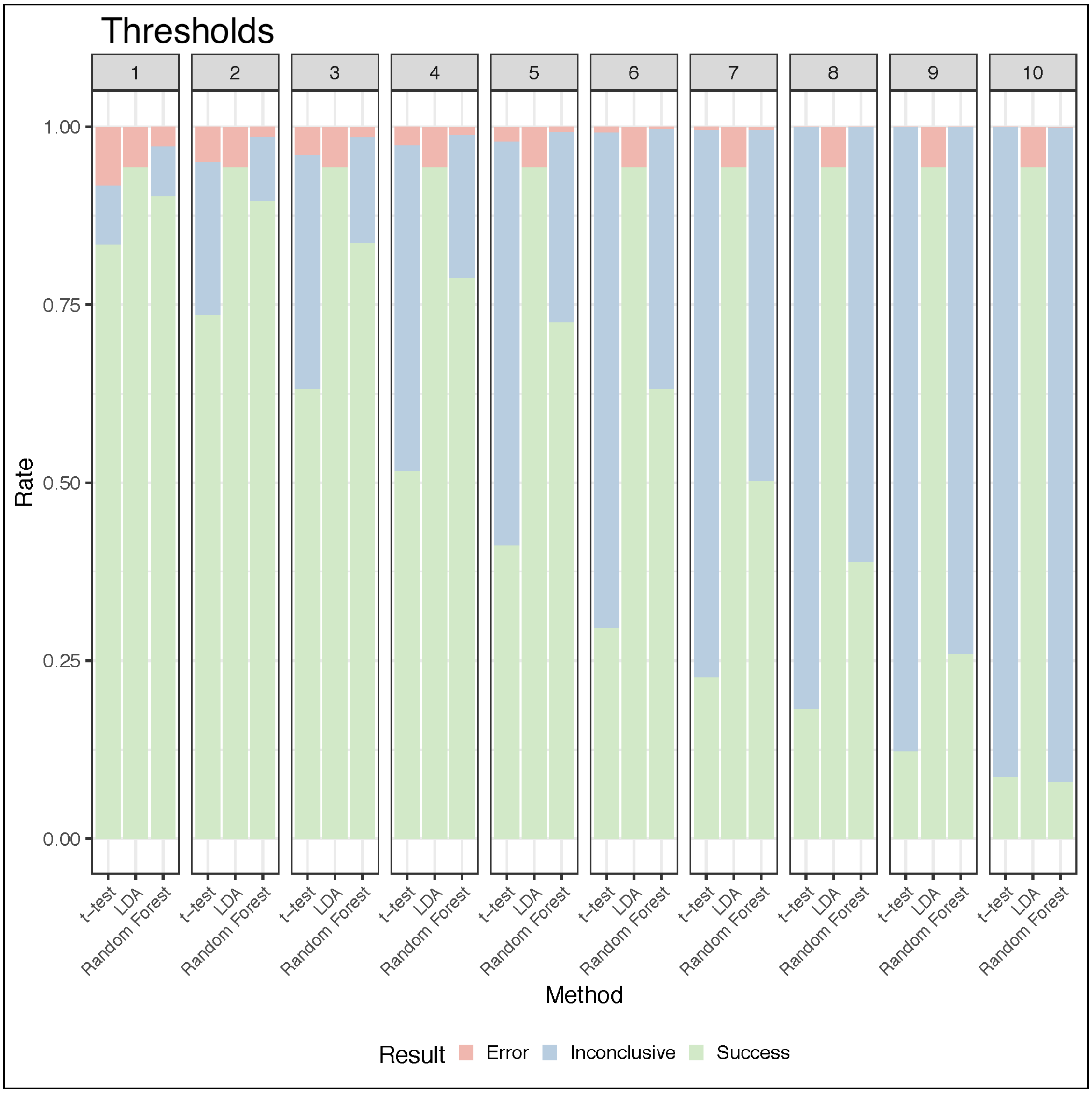

Figure S16: Comparison of success, inconclusive, and error rates for the three methods based on increasing thresholds for same day analysis of FA datasets. For t-test analysis, thresholds are the difference in the number of indistinguishable bins between the two most likely isomers. For LDA, the thresholds are the posterior probability of the classification, starting at 0.50 and increasing to 0.95, step-wise by 0.05. For Random Forest, the thresholds are the proportion of decision trees pointing to the classification, starting at 0.50 and increasing to 0.95, step-wise by 0.05. For LDA and Random Forest, ion current normalization was performed while the t-test used randomly assigned normalization per replicate.

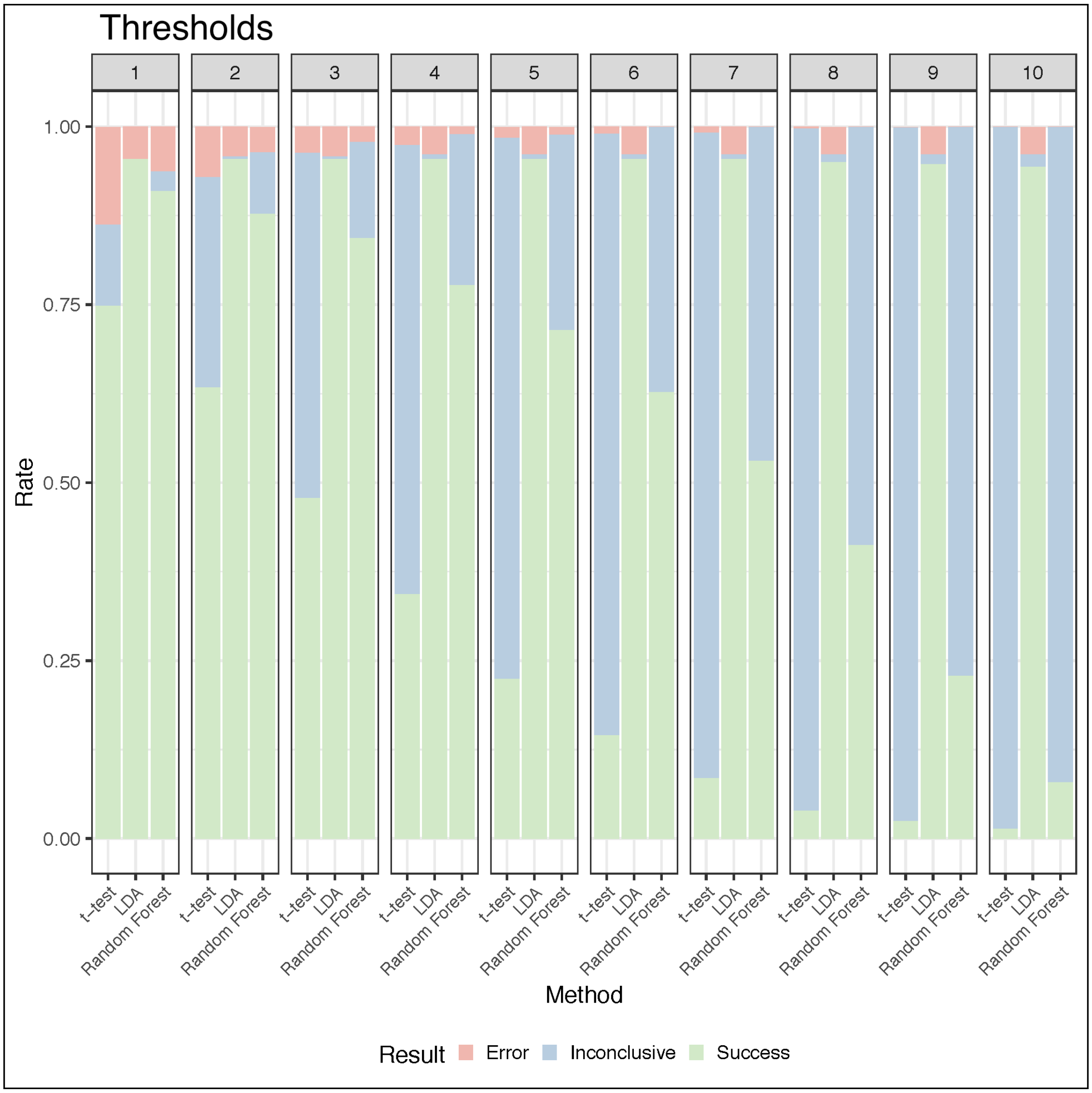

Figure S17: Comparison of success, inconclusive, and error rates for the three methods based on increasing thresholds for same day analysis of FMA datasets. For t-test analysis, thresholds are the difference in the number of indistinguishable bins between the two most likely isomers. For LDA, the thresholds are the posterior probability of the classification, starting at 0.50 and increasing to 0.95, step-wise by 0.05. For Random Forest, the thresholds are the proportion of decision trees pointing to the classification, starting at 0.50 and increasing to 0.95, step-wise by 0.05. For LDA and Random Forest, ion current normalization was performed while the t-test used randomly assigned normalization per replicate.

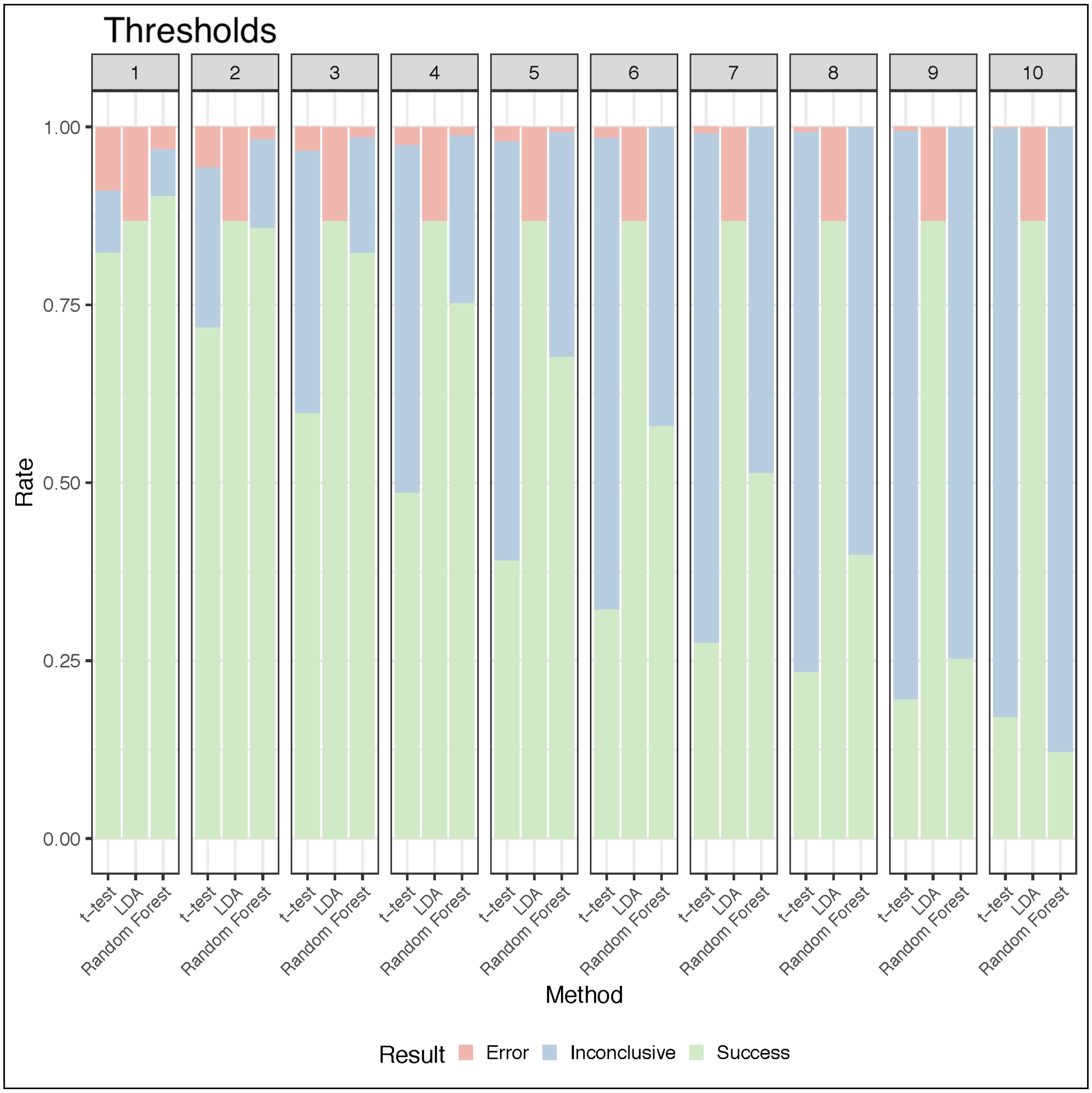

Figure S18: Comparison of success, inconclusive, and error rates for the three methods based on increasing thresholds for same day analysis of MMC datasets. For t-test analysis, thresholds are the difference in the number of indistinguishable bins between the two most likely isomers. For LDA, the thresholds are the posterior probability of the classification, starting at 0.50 and increasing to 0.95, step-wise by 0.05. For Random Forest, the thresholds are the proportion of decision trees pointing to the classification, starting at 0.50 and increasing to 0.95, step-wise by 0.05. For LDA and Random Forest, ion current normalization was performed while the t-test used randomly assigned normalization per replicate.

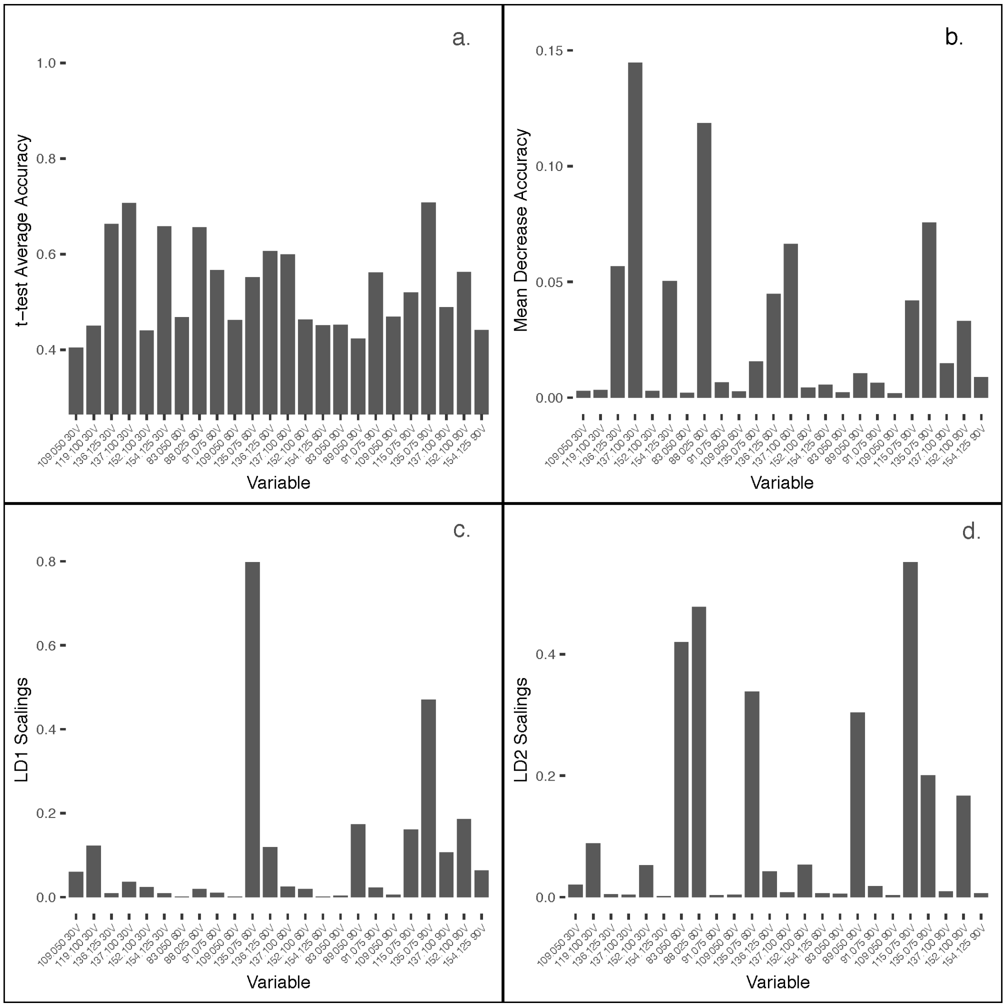

Figure S19: Comparison of variable importance for the three classification methods for the FA dataset using a 0.3% abundance threshold and ion current normalization (except for the Welch t-test which used a combination of normalization types across replicates). a) Welch t-test. Importance given in terms of average accuracy across 20 replicates, axis starts at 0.30% accuracy. b) Random Forest. Importance shown in terms of Mean Decrease Accuracy. c) Absolute values of the scalings for the first Linear Discriminant function, scaled to unit vector length d) Absolute values of the scalings for the second Linear Discriminant function, scaled to unit vector length.

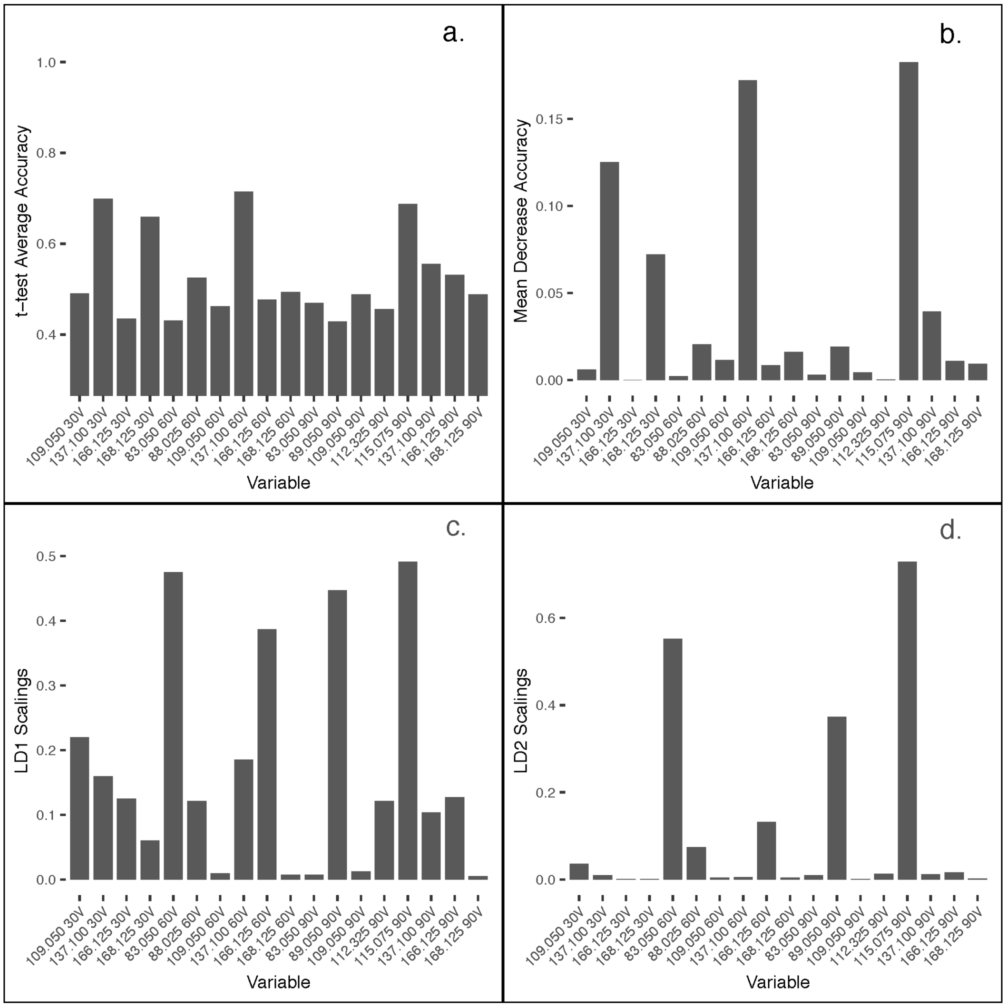

Figure S20: Comparison of variable importance for the three classification methods for the FMA dataset using a 0.3% abundance threshold and ion current normalization (except for the Welch t-test which used a combination of normalization types across replicates). a) Welch t-test. Importance given in terms of average accuracy across 20 replicates, axis starts at 0.30% accuracy. b) Random Forest. Importance shown in terms of Mean Decrease Accuracy. c) Absolute values of the scalings for the first Linear Discriminant function, scaled to unit vector length d) Absolute values of the scalings for the second Linear Discriminant function, scaled to unit vector length.

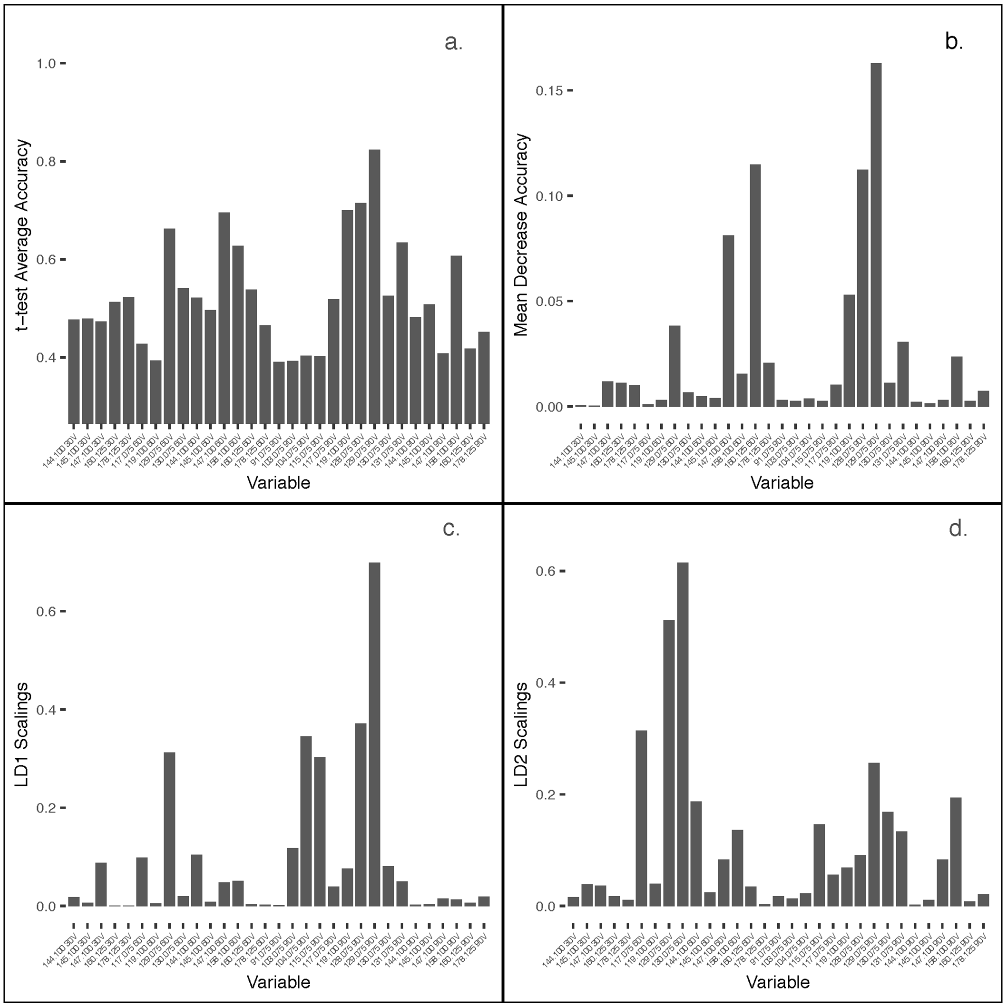

Figure S21: Comparison of variable importance for the three classification methods for the MMC dataset using a 1% abundance threshold and ion current normalization (except for the Welch t-test which used a combination of normalization types across replicates). a) Welch t-test. Importance given in terms of average accuracy across 20 replicates, axis starts at 0.30% accuracy. b) Random Forest. Importance shown in terms of Mean Decrease Accuracy. c) Absolute values of the scalings for the first Linear Discriminant function, scaled to unit vector length d) Absolute values of the scalings for the second Linear Discriminant function, scaled to unit vector length.
